# Anti-Lymphangiogenesis Components from Zoanthid *Palythoa tuberculosa*

**DOI:** 10.3390/md16020047

**Published:** 2018-01-31

**Authors:** Shu-Rong Chen, Shih-Wei Wang, Chien-Jung Su, Hao-Chun Hu, Yu-Liang Yang, Chi-Ting Hsieh, Chia-Chi Peng, Fang-Rong Chang, Yuan-Bin Cheng

**Affiliations:** 1 Graduate Institute of Natural Products, College of Pharmacy, Kaohsiung Medical University, Kaohsiung 807, Taiwan; highshorter@hotmail.com (S.-R.C.); shihwei@mmc.edu.tw (S.-W.W.); scj820826@gmail.com (C.-J.S.); drjcount@livemail.tw (H.-C.H.); ylyang@gate.sinica.edu.tw (Y.-L.Y.); 2 Department of Medicine, Mackay Medical College, New Taipei City 252, Taiwan; 3 Agricultural Biotechnology Research Center, Academia Sinica, Taipei 115, Taiwan; u98831001@gmail.com (C.-T.H.); helpme632@gmail.com (C.-C.P.); 4 Department of Marine Biotechnology and Resources, National Sun Yat-sen University, Kaohsiung 804, Taiwan; 5 Department of Medical Research, Kaohsiung Medical University Hospital, Kaohsiung 807, Taiwan

**Keywords:** pyrazines, alkaloids, *Palythoa tuberculosa*, anti-lymphangiogenesis

## Abstract

Three new compounds, tuberazines A–C (**1**–**3**), and eleven known compounds (**4**–**14**) were obtained from the ethanolic extract of Taiwanese zoanthid *Palythoa tuberculosa*. Compounds **1**–**4** are rare marine natural products with a pyrazine moiety, and compound **5** is a tricyclic tryptamine derivative isolated from nature for the first time. The structures of all isolated metabolites were determined by analyzing their IR, Mass, NMR, and UV spectrometric data. The absolute configuration of **1** was confirmed by comparing the trend of experimental electronic circular dichroism (ECD) with calculated ECD spectra. The anti-lymphangiogenic activities of new compounds were evaluated in human lymphatic endothelial cells (LECs). Of these, new compound **3** displayed the most potent anti-lymphangiogenesis property by suppressing cell growth and tube formation of LECs.

## 1. Introduction

Metastasis is a major cause of death in cancer patients. The metastatic spread of tumor cells to sentinel lymph nodes represents the crucial step of tumor dissemination in many types of human cancer [1]. Lymphangiogenesis, the formation of new lymphatic vessels from preexisting ones, is essential for the progression of cancer metastasis [2]. Therefore, lymphangiogenesis represents a potential therapeutic target for preventing lymphatic metastasis. The discovery of anti-lymphangiogeneic agents to suppress cancer metastasis is urgently needed.

Zoanthids are radially symmetrical cnidarians with two rows of tentacles, and they are usually found on the rocky coast of subtropical and tropical areas. The various colors of zoanthids are due to the symbiotic relationships with single-celled zooxanthellae, such as dinoflagellates. Due to their fascinating colors, these sessile benthic organisms are often used as ornamentals in aquaria. However, toxic substances produced by zoanthids sometimes cause cardiotoxicity, local itching, swelling, paralysis and necrosis to the keepers [3]. Accordingly, zoanthids are also regarded as rich sources of novel secondary metabolites with diverse bioactivities. For example, ecdysones isolated from *Palythoa mutuki* suppressed dengue virus production [4], while alkaloids purified from *Zoanthus kuroshio* inhibited superoxide anion generation and elastase release [5]. Compared to octocorals, the natural product investigation of zoanthids is relatively rare and we believe this type of marine invertebrate is a worthy research material. In our bioactive screening of Taiwanese zoanthids, the ethanolic extract of *P. tuberculosa* was found to have significant anti-cancer and anti-lymphangiogenic activities. Therefore, a series of bioassay-guided fractionations for this animal materials were carried out. The separation, structural elucidation, and bioactivities of three new and eleven known compounds from *P. tuberculosa* are herein reported.

## 2. Results

**Figure 1:**
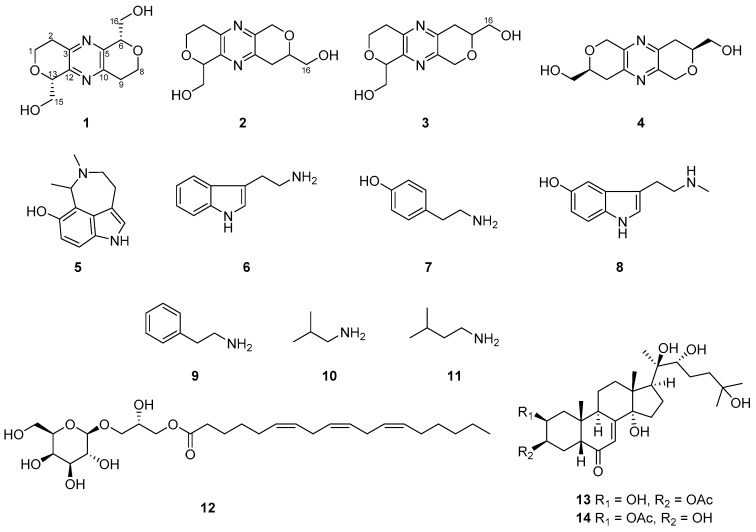
Structures of compounds **1**−**14**.

The zoanthid *P. tuberculosa* was collected from the northern seashore of Taiwan. The animal materials were extracted by ethanol three times and partitioned between EtOAc and H_2_O. The H_2_O layer was further partitioned between BuOH and H_2_O to give a relatively low polarity layer. Repeated column chromatography of the BuOH portion yielded three new compounds, tuberazines A–C (**1**–**3**), and six known compounds: palythazine (**4**) [6], 3,4,5,6-tetrahydro-7**-**hydroxy-5,6-dimethyl-1*H*-azepino[5,4,3-*cd*]indole (**5**) [7], tryptamine (**6**) [8], tyramine (**7**) [9], *N*-methylserotonin (**8**) [10], and phenethylamine (**9**) [11]. The EtOAc extract was also further partitioned between hexanes and 75% MeOH(aq) layers. Five known compounds [isobutylamine (**10**) [12], isoamylamine (**11**) [13], (2*S*)-2-hydroxy-3-[[(6*Z*,9*Z*,12*Z*)-1-oxo-6,9,12-octadecatrien-1-yl]oxy]propyl-β-d-galactopyranoside (**12**) [14], 20-hydroxyecdysone-3-acetate (**13**) [15], and 20-hydroxyecdysone-2-acetate (**14**) [15] were isolated from the 75% MeOH(aq) layer. Compounds **5**–**12** were obtained from the genus *Palythoa* for the first time. The structures of all the isolated compounds (**1**−**14**) are illustrated in Figure 1.

**Figure 2:**
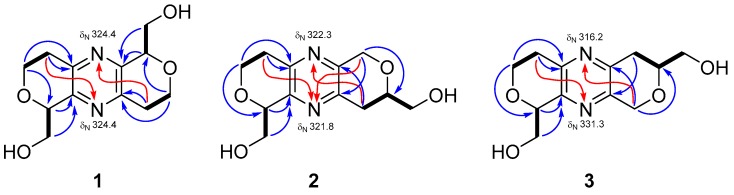
COSY (bold bond), ^1^H-^13^C HMBC (blue arrow), and ^1^H-^15^N HMBC (red arrow) correlations of **1**–**3**.

Tuberazine A (**1**), ${\left[\alpha \right]}_{D}^{26}$ +96 (*c* 0.05, MeOH), was isolated as white amorphous powder. The molecular formula of C_12_H_16_N_2_O_4_ and six degrees of unsaturation were inferred from its high-resolution electrospray ionisation mass spectrometry (HRESIMS) data (*m*/*z* 275.10028 [M + Na]^+^). In the infrared radiation (IR) spectrum of **1**, absorption at 3389 cm^−1^ revealed the existence of hydroxy functionality. The ultraviolet (UV) maximum absorption at 289 nm was similar to palythazine (**4**), suggesting these two compounds had the pyrazine framework. The ^1^H NMR data (Table 1) revealed the presences of one methylene (δ_H_ 2.84 and 3.15), two oxygen-bearing methylenes (δ_H_ 3.91 and 4.30; δ_H_ 3.98 and 4.06), and one oxymethine (δ_H_ 4.74). In the ^13^C NMR and distortionless enhancement by polarization transfer (DEPT) spectra of **1** (Table 2), six carbon signals can be classified into two aromatic nonprotonated carbons (δ_C_ 149.5 and 149.9), one aliphatic methylene (δ_C_ 32.4), two oxygen-bearing methylenes (δ_C_ 64.5 and 64.7), and one oxymethine (δ_C_ 79.8). Only of the half carbon and proton NMR signals was detected in comparison with the molecular formula of **1**, implying that **1** is a symmetric compound. In the COSY spectrum (Figure 2), correlations of H_2_-1 (δ_H_ 3.91 and 4.30)/H_2_-2 (δ_H_ 2.84 and 3.15) and H-13 (δ_H_ 4.74)/H_2_-15 (δ_H_ 3.98 and 4.06) were found. These two proton sequences were linked by virtue of the HMBC correlation (Figure 2) of H_2_-1/C-13 (δ_C_ 79.8). The deshielded chemical shifts of C-1 (δ_C_ 64.5) and C-13 (δ_C_ 79.8) suggested that an oxygen atom should be located between them. The HMBC correlations from H-13 and H-15 to C-12 (δc 149.9) indicated that C-13 and C-12 were connected. In addition, HMBC correlations from H_2_-1 and H_2_-2 to C-3 (δ_C_ 149.5) suggested the connection of C-2/C-3. Due to the lacks of direct linkages among nonprotonated carbons and protons in the ^1^H-^13^C HMBC spectrum, the existence of a pyrazine moiety (C-3, N-4, C-5, C-10, N-11, and C-12) was assured by comparing of the characteristic UV and NMR data with the congener, palythazine (**4**). However, two possible ^1^H and ^13^C symmetric structures (**1** and **1a**, Figure S22) matched the aforementioned 1D and 2D NMR data. In order to differentiate those structures, the ^1^H-^15^N HMBC experiment was executed using a high sensitivity CryoProbe NMR. In the ^1^H-^15^N HMBC spectrum, correlations from H_2_-2/H_2_-9 to N-4/N-11 (δ_N_ 324.4) were detected (Figure 2). These correlations are consistent with the ^4^*J*^1^_H_-^15^_N_ correlations reported for 3,5-dialkylpyridines [16]. The single nitrogen chemical shift revealed **1** was a nitrogen symmetric compound, which excluded the possibility of structure **1a**. Therefore, the planar structure of **1** was established.

**Figure 3:**
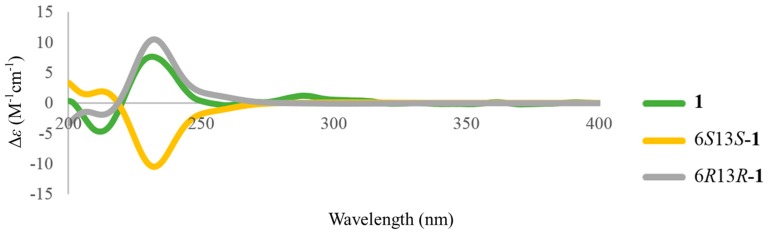
Calculated (yellow and gray lines) and experimental ECD (green line) spectra of **1**.

The absolute stereochemistry of **1** was determined by its optical rotation and comparing the experimental electronic circular dichroism (ECD) spectrum with the computer generated ECD spectra. The positive optical rotation value of **1** suggested compound **1** was optically active and was not a *meso* compound (6*R*13*S*-**1** or 6*S*13*R*-**1**). Thus, the ECD spectra of two stereoisomers 6*S*13*S*-**1** and 6*R*13*R*-**1** were calculated. The experimental ECD spectrum of **1** demonstrated positive Cotton effect at 232 nm and negative Cotton effect at 212 nm, which was consistent with the trend of the calculated ECD spectrum of 6*R*13*R*-**1** (Figure 3). From all of those spectroscopic data, structure **1** was unambiguously assigned to tuberazine A.

Tuberazine B (**2**) was obtained as white amorphous powder. The molecular formula C_12_H_16_N_2_O_4_ and six indices of hydrogen deficiency of **2** were determined by the sodiated ion peak at *m*/*z* 275.10025 in the HRESIMS. The IR absorption at 3387 cm^−1^ revealed the presence of hydroxy functionality. The pyrazine skeleton as **1** was deduced by the UV maximum absorptions at 287 and 212 nm. The UV, IR, ^1^H, and ^13^C NMR data of **2** (Table 1 and Table 2) were similar to those of **1**, which revealed that these two compounds were close related. The major difference between **2** and **1** was twelve carbon peaks were found in the ^13^C NMR spectrum of **2**, suggesting that **2** was an asymmetric compound. In the COSY spectrum, three proton sequences of H_2_-1 (δ_H_ 4.29 and 3.93)/H_2_-2 (δ_H_ 3.12 and 2.84), H-13 (δ_H_ 4.73)/H_2_-15 (δ_H_ 4.08 and 4.01), and H_2_-9 (δ_H_ 2.89)/H-8 (δ_H_ 3.93)/H_2_-16 (δ_H_ 3.74 and 3.69) were observed. The deshielded chemical shift of C-1 (δ_C_ 64.4) and C-13 (δ_C_ 79.7) together with the HMBC correlations from H_2_-1 to C-13 suggested the former two proton sequences were connected by an ether bridge. In addition, HMBC correlations from H_2_-2 to C-3 (δ_C_ 149.2) and from H-13 to C-12 (δ_C_ 150.3) revealed C-2 and C-13 were connected to the pyrazine moiety. The HMBC correlations from H_2_-6 (δ_H_ 4.84 and 4.76) to C-8 (δ_C_ 77.2), C-10 (δ_C_ 149.4) and from H_2_-9 to C-5 (δ_C_ 148.4) denoted C-5, C-6, O-7, C-8, C-9, and C-10 formed a hexacyclic ring. Considering the structures of congeners, palythazine and isopalythazine [17], one oxygen atom might locate on the position of 1 or 14 and the other on the position of 7 or 8. Therefore, four possible structures **2**, **2a**, **2b**, and **3** were proposed (Figure S23). The structure of **2** was also confirmed by the ^1^H-^15^N HMBC experiment. As a result, H_2_-2 and H_2_-6 showed ^4^*J* correlations to N-11 (δ_N_ 321.8), and H_2_-9 correlated to N-4 (δ_N_ 322.3). The same as **1**, the computer generated ECD spectra of **2** were proposed for determining its absolute stereochemistry. The calculated ECD spectra for isomers weren’t significantly different, so only the planar structure without stereochemistry is illustrated.

The molecular formula of tuberazine C (**3**), C_12_H_16_N_2_O_4_, was deduced from the pseudo-molecular ion peak at *m*/*z* 275.10023 [M + Na]^+^. The IR, UV, Mass, and NMR spectrometric data of **3** indicated it was a close analogue of **2**. In the COSY spectrum, cross-peaks of H_2_-1 (δ_H_ 4.30 and 3.93)/H_2_-2 (δ_H_ 3.15 and 2.83) and H-13 (δ_H_ 4.71)/H_2_-15 (δ_H_ 4.06 and 3.99) were found (Figure 2). These two proton sequences and the HMBC correlations of H_2_-1/C-13 (δ_C_ 79.7), H_2_-2/C-3 (δ_C_ 149.6), and H-13/C-12 (δ_C_ 149.9) were used to establish the dihydro-2*H*-pyran moiety connecting a pyrazine. On the other hand, the COSY correlations of H_2_-6 (δ_H_ 2.86)/H-7 (δ_H_ 3.91)/H_2_-16 (δ_H_ 3.73 and 3.69) together with the HMBC correlations of H_2_-9 (δ_H_ 4.85 and 4.76)/C-5 (δ_C_ 149.5), C-7 (δ_C_ 77.1) and H_2_-6/C-10 (δ_C_ 148.4) supported another dihydro-2*H*-pyran moiety attaching at the pyrazine. Therefore, compound **3** had the same possible structures (Figure S23) as those of **2**. The difference between **3** and **2** could be the oxygen atom locating in different position. This assumption was confirmed by the ^1^H-^15^N HMBC correlations from H_2_-2 (δ_H_ 3.15 and 2.83) to N-11 (δ_N_ 331.3) and from H_2_-9 (δ_H_ 4.85 and 4.76) to N-4 (δ_N_ 316.2). Hence, two oxygen atoms were placed in positions 8 and 14, and the structure of **3** was determined as shown.

**Figure 4:**
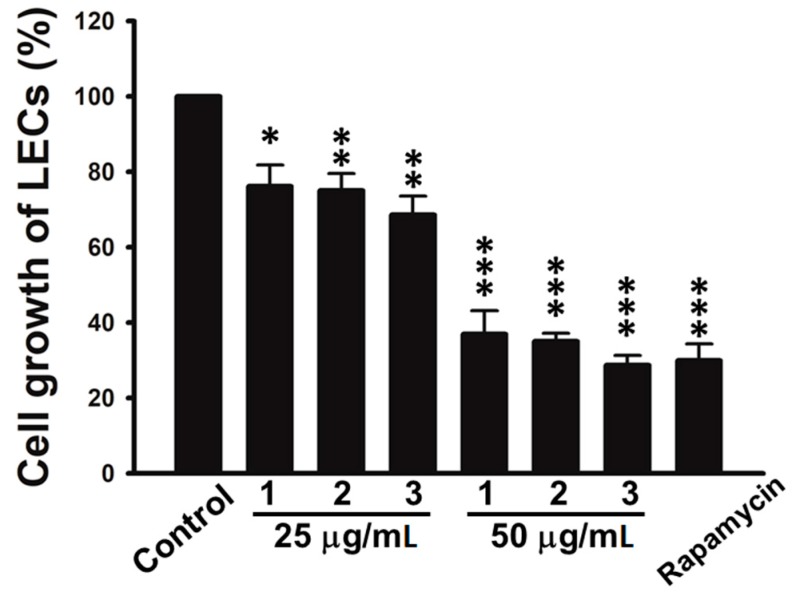
Effects of tuberazines A–C (**1**–**3**) on cell growth of human LECs. Cells were treated with tuberazines A–C (**1**–**3**) and rapamycin (5 μg/mL) for 48 h, and anti-lymphangiogeneic activity was determined using cell growth assay. Data are expressed as the mean ± SEM of five independent experiments. * *p* < 0.05, ** *p* < 0.01, *** *p* < 0.001 compared with the control group.

**Figure 5:**
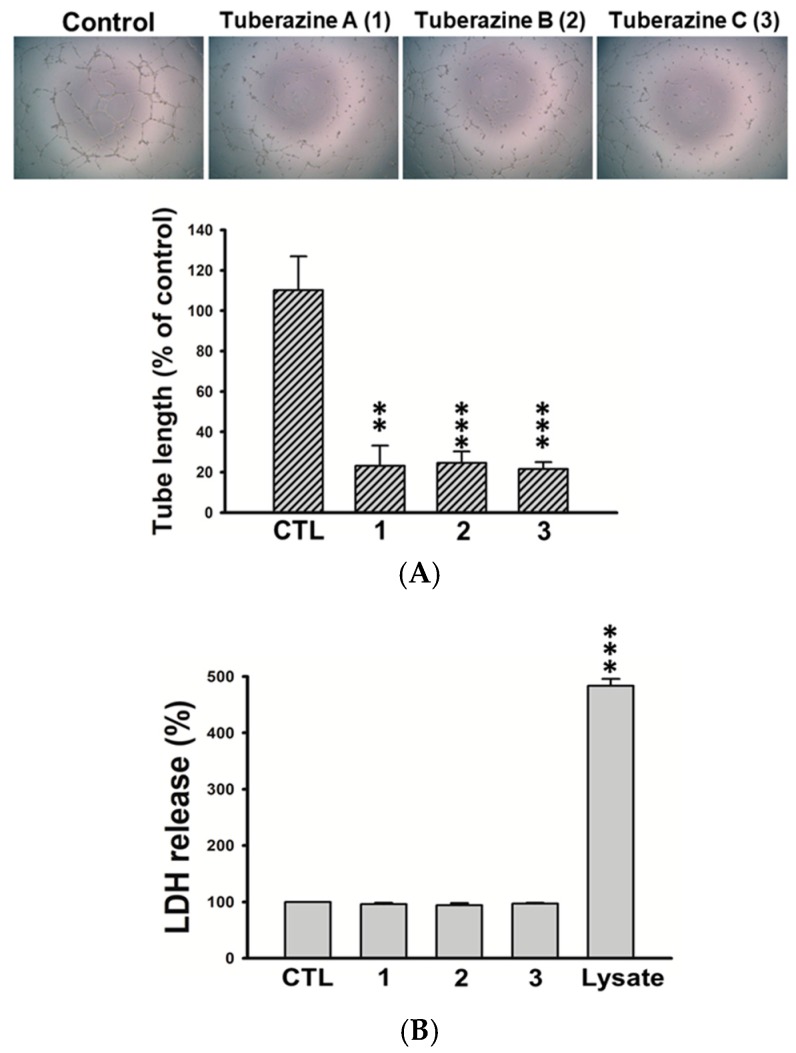
Effects of tuberazines A–C (**1**–**3**) on tube formation and cytotoxicity of human LECs. (**A**) Cells were plated on Matrigel-coated plates in tuberazines A–C (**1**–**3**) (50 μg/mL) for 8 h, and tubular morphogenesis was recorded by the inverted phase contrast microscope. Images were representative of results from five separate experiments. Tube formation was quantified by measuring the length of tubes with the use of Image-J. (**B**) Cells were treated with tuberazines A–C (**1**–**3**) (50 μg/mL) for 24 h, then the cytotoxicity was determined using LDH assay. Data are expressed as the mean ± SEM of at least three independent experiments. ** *p* < 0.01, *** *p* < 0.001 compared with the control group.

In this study, all isolated compounds were evaluated for their anti-cancer activities. The results showed that the isolates (**1**–**14**) exhibited no significant activities against three human cancer cell lines (A549, HepG2, and MDA-MB231). However, we found that tuberazines A–C (**1**–**3**) exerted promising anti-lymphangiogeneic activities in human lymphatic endothelial cells (LECs). Lymphangiogenesis has been shown to provoke tumor progression and lymphatic metastasis. Vascular endothelial growth factor-C (VEGF-C) is the most dominant lymphangiogenic factor, acting through VEGF receptor-3 (VEGFR-3) that is specifically expressed by LECs [18]. The activation of VEGF-C/VERFR-3 axis is responsible for LECs growth, migration and tube formation during lymphangiogenic process [2]. As shown in Figure 4, tuberazines A–C (**1**–**3**) inhibited cell growth of LECs in a concentration dependent manner. Tuberazines C (**3**) exhibited the most potent anti-lymphangiogeneic activity by acting the inhibitory effect on LECs growth (IC_50_ = 33 ± 1 μg/mL). Rapamycin, a well-known lymphangiogenesis inhibitor, was used as a positive control for in vitro anti-lymphangiogenesis assay (the IC_50_ value of compounds **1**–**3** and rapamycin are shown in Table S1). Capillary-like tubules are regarded as representative stage during lymphangiogenesis, we next performed tube formation assay to validate the anti-lymphangiogeneic effect of three new compounds (**1**–**3**) in human LECs. The results showed that tuberazines A–C (**1**–**3**) manifestly suppressed tube formation of LECs (Figure 5A). In addition, we found that tuberazines A–C (**1**–**3**) did not induce the significant lactate dehydrogenase (LDH) release in LECs (Figure 5B). Based upon these findings, we suggest that inhibitory activities of these compounds on lymphangiogenesis are not due to their cytotoxicity. Therefore, tuberazines A–C (**1**–**3**) are the promising marine products worthy of further development for impeding tumor lymphangiogenesis and metastasis.

## 3. Discussion

Marine natural products with pyrazine ring systems are quite rare. Only a few marine invertebrates were reported to produce pyrazine derivatives such as clavulazine [19], clavulazols [20], palythazine [17], barrenazines [21], botryllazines [22], and asteropterin [23]. Those compounds usually demonstrated diverse pharmacological properties. For example, barrenazines showed potent anti-tumor activity, which resulted in the development of total synthesis [24]. In this manuscript, three new dihydropyranopyrazine derivatives (**1**–**3**) were identified and their anti-lymphangiogeneic activity was first unveiled. Our findings not only extend the structural uniqueness of zoanthids, but provide further insight into pyrazine as an anti-lymphangiogeneic agent.

## 4. Materials and Methods

### 4.1. General Experimental Procedures

Perkin Elmer (Waltham, MA, USA) system 2000 FT-IR spectrophotometer was used for IR spectrum measurement. JASCO (Tokyo, Japan) P-1020 digital polarimeter was utilized for optical rotation measurement. UV and ECD spectra were recorded on JASCO V-530 UV/VIS spectrophotometer and JASCO J-815 CD spectrometer, respectively. Electrospray ionization (ESI) mass data were obtained from Waters (Milford, MA, USA) 2695 separations module and Bruker (Billerica, MA, USA) APEX II spectrometer (high resolution). NMR spectra were obtained by Bruker AVIII HD 700 MHz FT-NMR and AVAN CEIII 600 MHz FT-NMR. Merck (Darmstadt, Germany) silica gel 60 and GE Healthcare (Chicago, IL, USA) Sephadex LH-20 were used for column chromatography. The instrumentation for HPLC was composed of a Shimadzu (Kyoto, Japan) LC-20AD pump and a Shimadzu SPD-M20A PDA detector.

### 4.2. Animal Material

Specimens of *Palythoa tuberculosa* were collected in Taitung County, Taiwan, in April 2016. The research samples were identified by Dr. Yuan-Bin Cheng. A voucher specimen (no. KMU-PT1) was deposited in the Graduate Institute of Natural Products, College of Pharmacy, Kaohsiung Medical University.

### 4.3. Extraction and Isolation

The animal materials were lyophilized and the dry materials were extracted by ethanol three times. The ethanolic extract was partitioned between ethyl acetate and water (1:1) to afford two different polarity layers. The water layer was further partitioned by *n*-butanol and water (1:1) to give an *n*-butanol soluble extract. This extract (3.2 g) was chromatographed by a Sephadex LH-20 column eluted with methanol to give five fractions (A–E). Fraction D (958.0 mg) was separated by a Si gel open column stepwise eluted with dichloromethane and methanol (30:1 to 0:1) to furnish seven fractions (D1–D7). Fraction D1 (46.6 mg) was isolated by RP-HPLC (phenyl-hexyl, 15% methanol, isocratic elution) to afford compound **1** (1.5 mg). Fraction D-3 (40.6 mg) was purified by RP-HPLC (phenyl-hexyl, 20% methanol, isocratic elution) to yield compounds **2** (2.3 mg), **3** (2.3 mg) and **5** (12.4 mg). Fraction D6 (30.1 mg) was separated by RP-HPLC (C_18_, 5% to 100% methanol, gradient elution) to give compounds **4** (1.5 mg) and **9** (5.8 mg). Fraction D7 (230.4 mg) was chromatographed by a Si gel column stepwise eluted with dichloromethane and methanol (10:1 to 0:1) to furnish nine fractions (D7A–D7I). Fraction D7H (87.3 mg) was isolated by RP-HPLC (C_18_, 5% to 95% acetonitrile, gradient elution) to give compound **6** (8.4 mg). Fraction D7I (36.2 mg) was purified by RP-HPLC (C_18_, 1% methanol, isocratic elution) to yield compounds **7** (1.9 mg) and **8** (2.1 mg). On the other hand, the ethyl acetate was subsequently partitioned between *n*-hexane and 75% methanol. The 75% methanolic layer (4.1 g) was subjected to a Sephadex LH-20 column eluted with methanol to afford fractions F–I. Fraction G (4.1 g) was isolated by a Si gel open column stepwise eluted with dichloromethane and methanol (34:1 to 5:1) to give fractions G1–G6. Fraction G2 (255.8 mg) was purified by another Si gel column isocratic eluted with ethyl acetate and methanol (6:1) to give subfractions G2A–G2C. Subfraction G2B (16.7 mg) was isolated by RP-HPLC (phenyl-hexyl, 25% to 55% acetonitrile, gradient elution) to give compounds **13** (0.5 mg) and **14** (0.6 mg). Subfraction G2C (42.6 mg) was purified by RP-HPLC (phenyl-hexyl, 50% acetonitrile, isocratic elution) to give compound **12** (6.3 mg). Fraction G3 (357.9 mg) was separated by Si gel column isocratic eluted with dichloromethane and methanol (9:1) to afford subfractions G3A–G3E. Subfraction G3D (58.6 mg) was further isolated by RP-HPLC (phenyl-hexyl, 20% acetonitrile, isocratic elution) to yield compounds **10** (2.7 mg) and **11** (12.0 mg).

Tuberazine A (**1**): colorless amorphous powder; ${\left[\alpha \right]}_{D}^{26}$ +96 (*c* 0.05, MeOH); UV (MeOH) *λ*_max_ (log ε) 289 (4.04), 212 (4.23) nm; ECD (MeOH) *λ*_max_ (Δε): 212 (−4.65), 232 (+7.64) nm; IR (ATR) *ν*_max_: 3389, 2972, 2920, 1685, 1520, 1086, 882 cm^−1^; ^1^H NMR and ^13^C NMR data, see Table 1 and Table 2; HRESIMS *m*/*z* 275.10028 [M + Na]^+^ (calcd. for C_12_H_16_N_2_O_4_Na, 275.10023).

Tuberazine B (**2**): colorless amorphous powder; ${\left[\alpha \right]}_{D}^{26}$ +22 (*c* 0.05, MeOH); UV (MeOH) *λ*_max_ (log ε) 287 (4.08), 212 (4.12) nm; ECD (MeOH) *λ*_max_ (Δε): 215 (−1.27), 319 (+0.89) nm; IR (ATR) *ν*_max_: 3387, 2974, 2916, 1657, 1231, 1091, 1085, 884 cm^−1^; ^1^H NMR and ^13^C NMR data, see Table 1 and Table 2; HRESIMS *m*/*z* 275.10025 [M + Na]^+^ (calcd. for C_12_H_16_N_2_O_4_Na, 275.10023).

Tuberazine C (**3**): colorless amorphous powder; ${\left[\alpha \right]}_{D}^{26}$ +45 (*c* 0.05, MeOH); UV (MeOH) *λ*_max_ (log ε) 288 (4.04), 213 (4.23) nm; ECD (MeOH) *λ*_max_ (Δε): 214 (−0.95), 291 (+1.11) nm; IR (ATR) *ν*_max_: 3387, 2922, 1648, 1407, 1082, 1059, 876 cm^−1^; ^1^H NMR and ^13^C NMR data, see Table 1 and Table 2; HRESIMS *m*/*z* 275.10014 [M + Na]^+^ (calcd. for C_12_H_16_N_2_O_4_Na, 275.10023).

### 4.4. Ecd Calculations

The lowest energies of 6*S*13*S*-**1** and 6*R*13*R*-**1** were calculated and the data were performed by the Gaussian 09 software (Gaussian Inc., Wallingford, CT, USA). The density functional theory (DFT) at the B3LYP/6-31G(d) level in the gas phase were used to obtain the restricted conformation. The minima energies of 20 conformers were computed by the time-dependent density functional theory (TDDFT) methodology at the B3LYP/6-311++G(d,p) level. The final ECD files were generated by GaussSum 2.2.5 software with a bandwidth σ of 0.5 eV. The calculated ECD and experimental ECD curves were drawn by Excel.

### 4.5. Cell Culture of Human LECs

The human telomerase-immortalized human dermal lymphatic endothelial cells (hTERT-HDLECs), an immortalized human LEC line, was purchased from Lonza (Walkersville, MD, USA). The cultivation and maintenance of human LECs were performed as described previously [25]. Briefly, LECs were grown in EGM-2MV BulletKit medium consisting of EBM-2 basal medium plus SingleQuots kit (Lonza, Basel, Switzerland). Cells were seeded onto 1% gelatin-coated plastic ware and cultured at 37 °C with 5% CO_2_ for further treatment. Experiments were conducted on LECs between passages 10 and 20.

### 4.6. Cell Growth Assay

LECs were seeded onto 96-well plates in at the density of 5 × 10^3^ cells per well. After 24 h incubation, the culture medium was removed and cells were treated with EGM-2MV BulletKit medium in the absence or presence of tested compounds for 48 h. Then, cells were fixed with 50% TCA to terminate reaction, and 0.4% SRB (Sigma, St. Louis, MO, USA) in 1% acetic acid was added to each well. After a 15-min incubation, the plates were washed, and dye was dissolved by 10 mM Tris buffer. The 96-well plate was read by enzyme-linked immunosorbent assay (ELISA) reader (515 nm) to get the absorbance density values.

### 4.7. Capillary Tube Formation Assay

Matrigel (50 μL) was added to 96-well plates for determining the differentiation of LECs into a capillary tube-like structure. Matrigel-coated 96-well plates were incubated at 37 °C for 30 min to allow for polymerization. After gel formation, LECs were seeded per well at a density of 2 × 10^4^/200 μL in EGM-2MV BulletKit medium with the indicated concentration of tested compounds. After 8 h incubation, LECs tube formation were taken with the inverted phase contrast microscope. The number of tube branches and total tube length were calculated using the MacBiophotonics Image J software.

### 4.8. Cytotoxicity Assay

LECs were seeded onto 96-well plates at the density of 5 × 10^3^ cells per well. After 24 h incubation, cells were treated with the EGM-2MV BulletKit medium with the indicated concentration of tested compounds. Then, the percentage of LDH release in the collected medium was determined by non-radioactive cytotoxicity assay kit (Promega, Madison, WI, USA).

### 4.9. Anti-Cancer Assay

A549, HepG2, and MDA-MB-231 cells were cultured on 96-well plate at the density of 5 × 10^3^ cells per well. Tested compound were afterwards added for 72 h. The cell viability was determined by the MTT assay. Doxorubicin and paclitaxel were used as positive controls. ELISA reader (Thermo Electron Cooperation, Waltham, MA, USA) was used for absorbance (550 nm) measurement.

### 4.10. Statistical Analysis

Data are presented as the mean ± SEM for the indicated number of separate experiment. Statistical analyses of data were performed with one-way ANOVA followed by Student’s *t*-test, and *p*-values less than 0.05 were considered significant.

## Figures and Tables

**Table 1:** ^1^H NMR Data of **1**–**3** in CD_3_OD *^a^*.

NO.	1	2	3
1	4.30, ddd (11.7, 5.7, 2.9)	4.29, ddd (11.6, 5.6, 3.5)	4.30, ddd (11.9, 5.6, 4.2)
	3.91, ddd (11.7, 10.5, 3.5)	3.93, ddd (11.6, 10.2, 3.5)	3.93, ddd (11.9, 9.8, 4.2)
2	3.15, ddd (16.8, 10.5, 5.6)	3.12, ddd (16.8, 10.2, 5.6)	3.15, ddd (16.8, 9.8, 5.6)
	2.84, dt (16.8, 3.5)	2.84, dt (16.8, 3.5)	2.83, m
6	4.74, m	4.84, d (16.1)	2.86, m
	-	4.76, d (16.1)	-
7	-	-	3.91, ddd (11.9, 9.8, 4.2)
8	4.30, ddd (11.7, 5.6, 3.5)	3.93, ddd (11.2, 6.3, 3.5)	-
	3.91, ddd (11.7, 10.5, 3.5)	-	-
9	3.15, ddd (16.8, 10.5, 5.6)	2.89, m	4.85, d (14.0)
	2.84, dt (16.8, 3.5)	-	4.76, d (14.0)
13	4.74, m	4.73, m	4.71, m
15	4.06, dd (11.8, 2.7)	4.08, dd (11.9, 2.8)	4.06, dd (11.9, 2.8)
	3.98, dd (11.8, 5.7)	4.01, dd (11.9, 5.6)	3.99, dd (11.9, 5.6)
16	4.06, dd (11.8, 2.7)	3.74, dd (11.2, 3.5)	3.73, dd (11.9, 3.8)
	3.98, dd (11.8, 5.7)	3.69, dd (11.2, 6.3)	3.69, dd (11.9, 6.3)

*^a^* Data were measured at 700 MHz; Chemical shifts are in ppm. *J* values (Hz) in parentheses.

**Table 2:** ^13^C NMR Data of **1**–**3** in CD_3_OD *^a^*.

NO.	1	2	3
1	64.5, CH_2_	64.4, CH_2_	64.4, CH_2_
2	32.4, CH_2_	33.2, CH_2_	33.2, CH_2_
3	149.5, C	149.2, C	149.6, C
5	149.9, C	148.4, C	149.5, C
6	79.8, CH	69.6, CH_2_	33.5, CH_2_
7	-	-	77.1, CH
8	64.5, CH_2_	77.2, CH	-
9	32.4, CH_2_	33.5, CH_2_	69.6, CH_2_
10	149.5, C	149.4, C	148.4, C
12	149.9, C	150.3, C	149.9, C
13	79.8, CH	79.7, CH	79.7, CH
15	64.7, CH_2_	64.6, CH_2_	64.5, CH_2_
16	64.7, CH_2_	65.5, CH_2_	65.5, CH_2_

***^a^*** Data were measured at 175 MHz; Chemical shifts are in ppm.

## Supplementary Materials

The following are available online at http://www.mdpi.com/1660-3397/16/2/47/s1, 1D and 2D NMR data of compounds **1**–**3**. Possible structures of compounds **1** and **2**.

## References

[1] Trappen P.O.V., Pepper M.S. (2002). Lymphatic dissemination of tumour cells and the formation of micrometastases. Lancet Oncol..

[2] Stacker S.A., Williams S.P., Karnezis T., Shayan R., Fox S.B., Achen M.G. (2014). Lymphangiogenesis and lymphatic vessel remodelling in cancer. Nat. Rev. Cancer.

[3] Hoffmann K., Hermanns-Clausen M., Buhl C., Büchler M.W., Schemmer P., Mebs D., Kauferstein S. (2008). A case of palytoxin poisoning due to contact with zoanthid corals through a skin injury. Toxicon.

[4] Lee J.-C., Chang F.-R., Chen S.-R., Wu Y.-H., Hu H.-C., Wu Y.-C., Backlund A., Cheng Y.-B. (2016). Anti-dengue virus constituents from formosan zoanthid *Palythoa mutuki*. Mar. Drugs.

[5] Hsu Y.-M., Chang F.-R., Lo I.-W., Lai K.-H., El-Shazly M., Wu T.-Y., Du Y.-C., Hwang H.-Y., Cheng Y.-B., Wu Y.-C. (2016). Zoanthamine-Type alkaloids from the zoanthid *Zoanthus kuroshio* collected in Taiwan and their effects on inflammation. J. Nat. Prod..

[6] Brehm M., Göckel V.H., Jarglis P., Lichtenthaler F.W. (2008). Expedient conversion of d-glucose into 1,5-anhydro-d-fructose and into single stereogenic-center dihydropyranones, suitable six-carbon scaffolds for concise syntheses of the soft-coral constituents (−)-bissetone and (−)-palythazine. Tetrahedron Asymmetry.

[7] Somei M., Teranishi S., Yamada K., Yamada F. (2001). The chemistry of indoles. CVII. a novel synthesis of 3,4,5,6-tetrahydro-7-hydroxy-1*H*-azepino[5,4,3-*cd*]indoles and a new finding on Pictet-Spengler reaction. Chem. Pharm. Bull..

[8] Carvalho M.A., Arruda E.G.R., Profirio D.M., Gomes A.F., Gozzo F.C., Formiga A.L.B., Corbi P.P. (2015). Chemical and spectroscopic characterizations, ESI-QTOF mass spectrometric measurements and DFT studies of new complexes of palladium (II) with tryptamine and mefenamic acid. J. Mol. Struct..

[9] Sun J.F., Wu Y., Yang B., Liu Y. (2015). Chemical constituents of marine sponge *Halichondria* sp. from south China sea. Chem. Nat. Compd..

[10] Somei M., Teranishi S., Yamada K., Yamada F. (2001). The chemistry of indoles. CIII. simple syntheses of serotonin, *N*-methylserotonin, bufotenine, 5-methoxy-*N*-methyltryptamine, bufobutanoic acid, *N*-(indol-3-yl)methyl-5-methoxy-*N*-methyltryptamine, and lespedamine based on 1-hydroxyindole chemistry. Chem. Pharm. Bull..

[11] Jiang H., Hu J., Xu X., Zhou Y. (2015). Preparation and characterization of primary amines by potassium borohydride-copper chloride system from nitriles. Asian J. Chem..

[12] Katritzky A.R., Yousaf T.I., Chen B.C. (1986). An H-1, C-13 and N-15 NMR study of the paal-knorr condensation of acetonylacetone with primary amines. Tetrahedron.

[13] Rabenstein D.L., Sayer T.L. (1976). Carbon-13 chemical shift parameters for amines, carboxylic acids, and amino acids. J. Magn. Reson..

[14] Herrero M., Vicente M.J., Cifuentes A., Ibáñez E. (2007). Characterization by high-performance liquid chromatography/electrospray ionization quadrupole time-of-flight mass spectrometry of the lipid fraction of *Spirulina platensis* pressurized ethanol extract. Rapid Commun. Mass Spectrom..

[15] Suksamrarn A., Pattanaprateep P. (1995). Selective acetylation of 20-hydroxyecdysone partial synthesis of some minor ecdysteroids and analogues. Tetrahedron.

[16] Kline M., Cheatham S. (2003). A robust method for determining ^1^H-^15^N long-range correlations: ^15^N optimized CIGAR-HMBC experiments. Mag. Reson. Chem..

[17] Uemura D., Toya Y., Watanabe I., Hirata Y. (1979). Isolation and structures of two new pyrazines, palythazine and isopalythazine from *Palythoa tuberculosa*. Chem. Lett..

[18] Wissmann C., Detmar M. (2006). Pathways targeting tumor lymphangiogenesis. Clin. Cancer Res..

[19] Watanabe K., Iguchi K., Fujimori K. (1998). Clavulazine, a new marine pyrazine congener from the Okinawan soft coral Clavularia viridis. Hetercycles.

[20] Shen Y.-C., Wang L.-T., Cheng Y.-B., Khalil A.T., Chen M.-H., Lin Y.-C. (2004). Clavulazols A and B, two new pyrazine derivatives from *Clavularia viridis*. J. Chin. Chem. Soc..

[21] Chill L., Aknin M., Kashman Y. (2003). Barrenazine A and B; two new cytotoxic alkaloids from an unidentified tunicate. Org. Lett..

[22] Durán R., Zubiv E., Ortega M.J., Naranjo S., Salvá J. (1999). Novel alkaloids from the red ascidian *Botryllus leachi*. Tetrahedron.

[23] Murayama S., Nakao Y., Matsunaga S. (2008). Asteropterin, an inhibitor of cathepsin B, from the marine sponge *Asteropus simplex*. Tetrahedron Lett..

[24] Focken T., Charette A.B. (2006). Stereoselective synthesis of pyridinones: Application to the synthesis of (−)-barrenazines. Org. Lett..

[25] Wang L.-H., Lin C.-Y., Liu S.-C., Liu G.-T., Chen Y.-L., Chen J.-J., Chan C.-H., Lin T.-Y., Chen C.-K., Xu G.-H. (2016). CCL5 promotes VEGF-C production and induces lymphangiogenesis by suppressing miR-507 in human chondrosarcoma cells. Oncotarget.

