# Supplementary material for: Anti-Lymphangiogenesis Components from Zoanthid Palythoa tuberculosa
Source: Mar Drugs. 2018 Jan 31;16(2):47. doi: 10.3390/md16020047 (PMC5852475; doi:10.3390/md16020047)
Supplement: Supplementary file 1 [file marinedrugs-16-00047-s001.pdf]

Supporting Information for

Anti-lymphangiogenesis Components from Taiwanese Zoanthid *Palythoa tuberculosa*

Shu-Rong Chen [1,†], Shih-Wei Wang [1,2,†], Chien-Jung Su [1], Hao-Chun Hu [1], Yu-Liang Yang [1,3], Chi-Ting Hsieh [3], Chia-Chi Peng [3], Fang-Rong Chang [1,4,*] and Yuan-Bin Cheng [1,5,*]

[1] Graduate Institute of Natural Products, College of Pharmacy, Kaohsiung Medical University, Kaohsiung 807, Taiwan

[2] Department of Medicine, Mackay Medical College, New Taipei City 252, Taiwan

[3] Agricultural Biotechnology Research Center, Academia Sinica, Taipei 115, Taiwan

[4] Department of Marine Biotechnology and Resources, National Sun Yat-sen University, Kaohsiung 804, Taiwan

[5] Department of Medical Research, Kaohsiung Medical University Hospital, Kaohsiung 807, Taiwan

* Correspondence: jmb@kmu.edu.tw; Tel.: +886-7-312-1101 (ext. 2197); aaronfrc@kmu.edu.tw; Tel.: +886-7-312-1101 (ext. 2162)

† These authors contributed equally to this work.

Table of Contents

Figure S1. ^{1}H NMR spectrum of tuberazine A (**1**) (CD_3OD, 700 MHz) 2
Figure S2. ^{13}C NMR spectrum of tuberazine A (**1**) (CD_3OD, 175 MHz) 3
Figure S3. COSY spectrum of tuberazine A (**1**) 4
Figure S4. HSQC spectrum of tuberazine A (**1**) 5
Figure S5. HMBC spectrum of tuberazine A (**1**) 6
Figure S6. ^{1}H-^{15}N HMBC spectrum of tuberazine A (**1**) 7
Figure S7. ^{1}H NMR spectrum of tuberazine B (**2**) (CD_3OD, 700 MHz) 8
Figure S8. ^{13}C NMR spectrum of tuberazine B (**2**) (CD_3OD, 175 MHz) 9
Figure S9. COSY spectrum of tuberazine B (**2**) 10
Figure S10. HSQC spectrum of tuberazine B (**2**) 11
Figure S11. HMBC spectrum of tuberazine B (**2**) 12
Figure S12. ^{1}H-^{15}N HMBC spectrum of tuberazine B (**2**) 13
Figure S13. ^{1}H NMR spectrum of tuberazine C (**3**) (CD_3OD, 700 MHz) 14
Figure S14. ^{13}C NMR spectrum of tuberazine C (**3**) (CD_3OD, 175 MHz) 15
Figure S15. COSY spectrum of tuberazine C (**3**) 16
Figure S16. HSQC spectrum of tuberazine C (**3**) 17
Figure S17. HMBC spectrum of tuberazine C (**3**) 18
Figure S18. ^{1}H-^{15}N HMBC spectrum of tuberazine C (**3**) 19
Figure S19. HRESIMS spectrum of tuberazine A (**1**) 20
Figure S20. HRESIMS spectrum of tuberazine B (**2**) 21
Figure S21. HRESIMS spectrum of tuberazine C (**3**) 22
Figure S22. Possible structures of **1** 23
Figure S23. Possible structures of **2** 23
Table S1. Anti-lymphangiogenic activity of selected compounds 24

Figure S1. ^{1}H NMR spectrum of tuberazine A (**1**) (CD_3OD, 700 MHz)

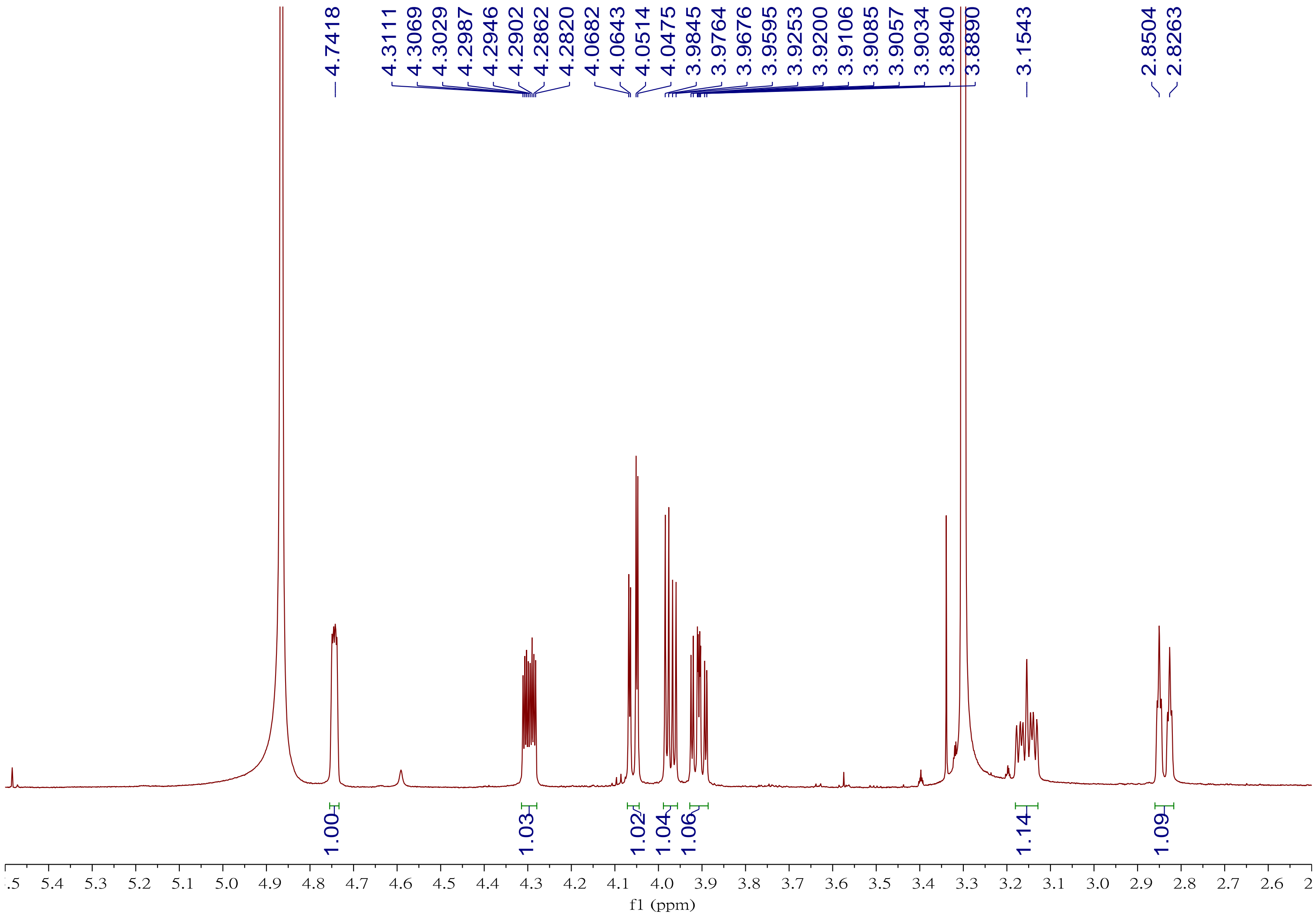

Figure S2. ^{13}C NMR spectrum of tuberazine A (**1**) (CD$_3$OD, 175 MHz)

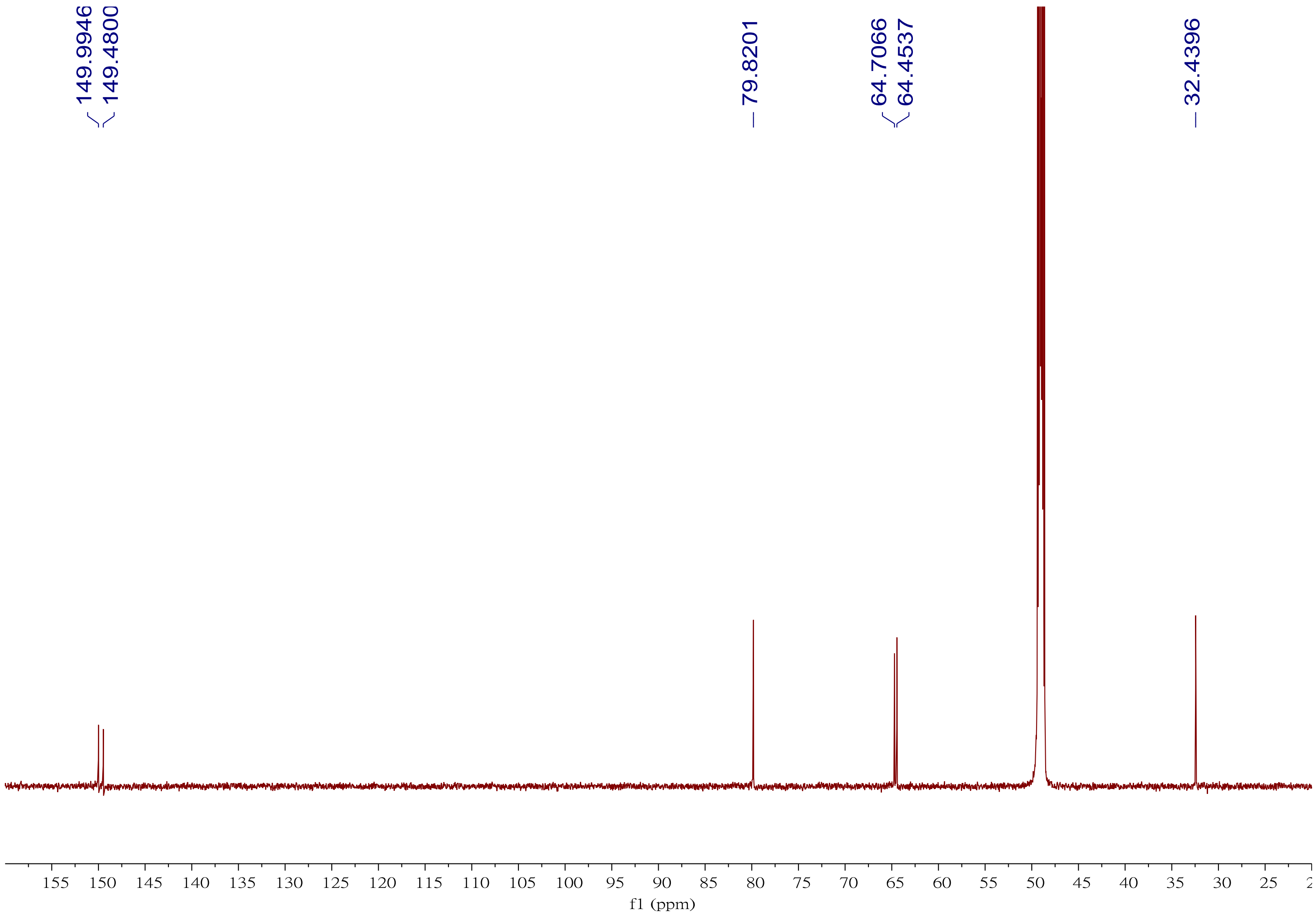

Figure S3. COSY spectrum of tuberazine A (**1**)

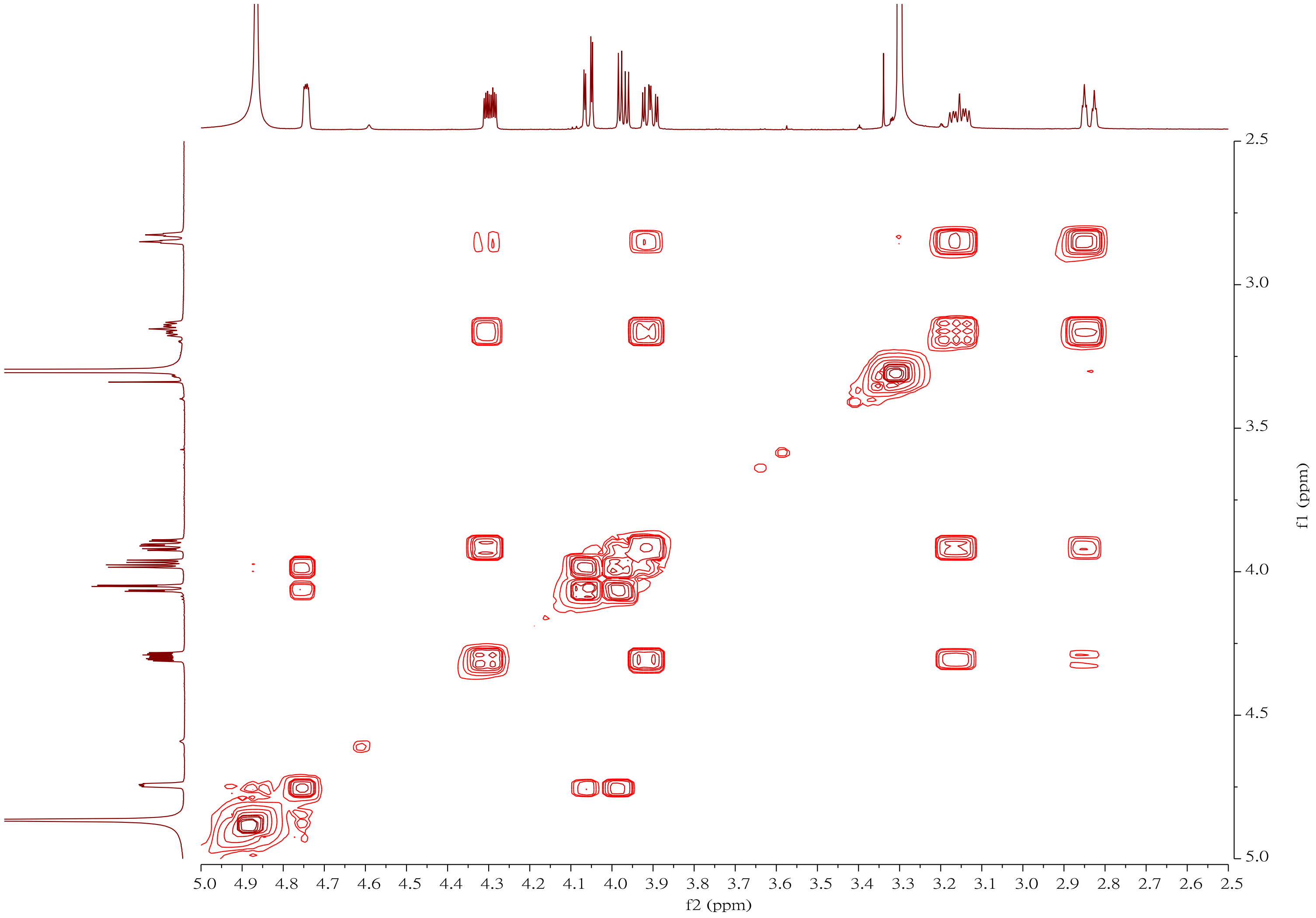

Figure S4. HSQC spectrum of tuberazine A (**1**)

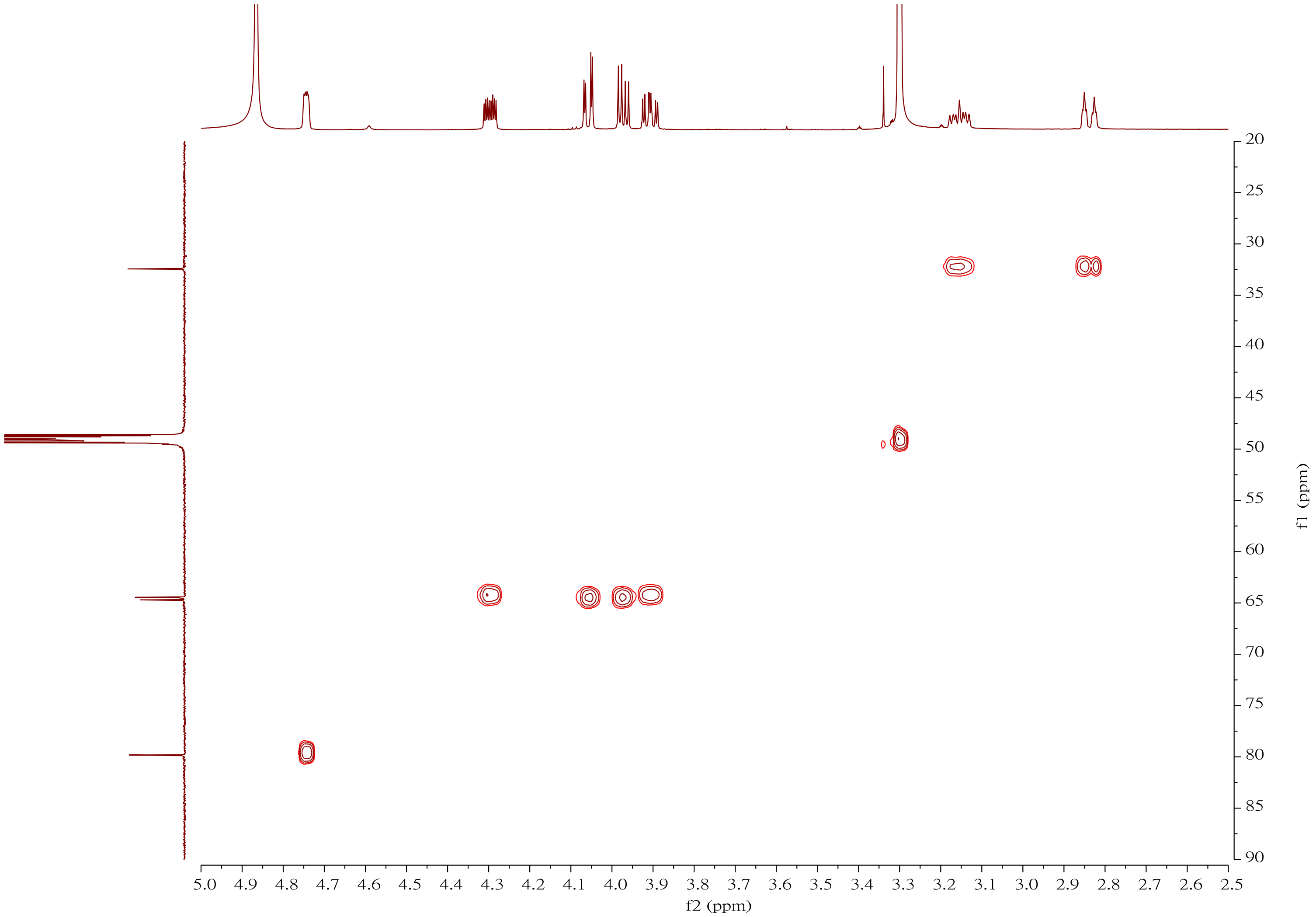

Figure S5. HMBC spectrum of tuberazine A (**1**)

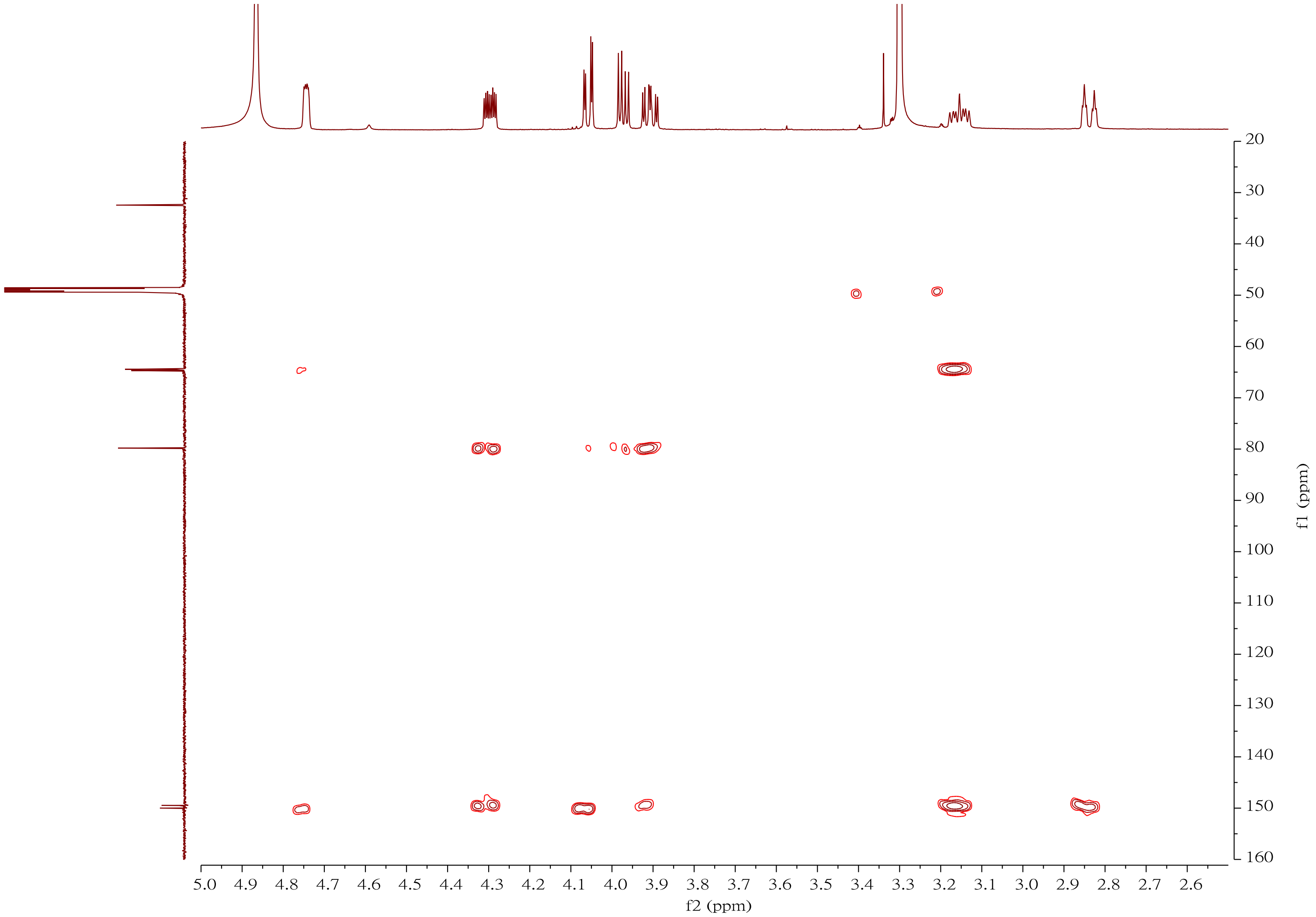

Figure S6. ^{1}H-^{15}N HMBC spectrum of tuberazine A (**1**)

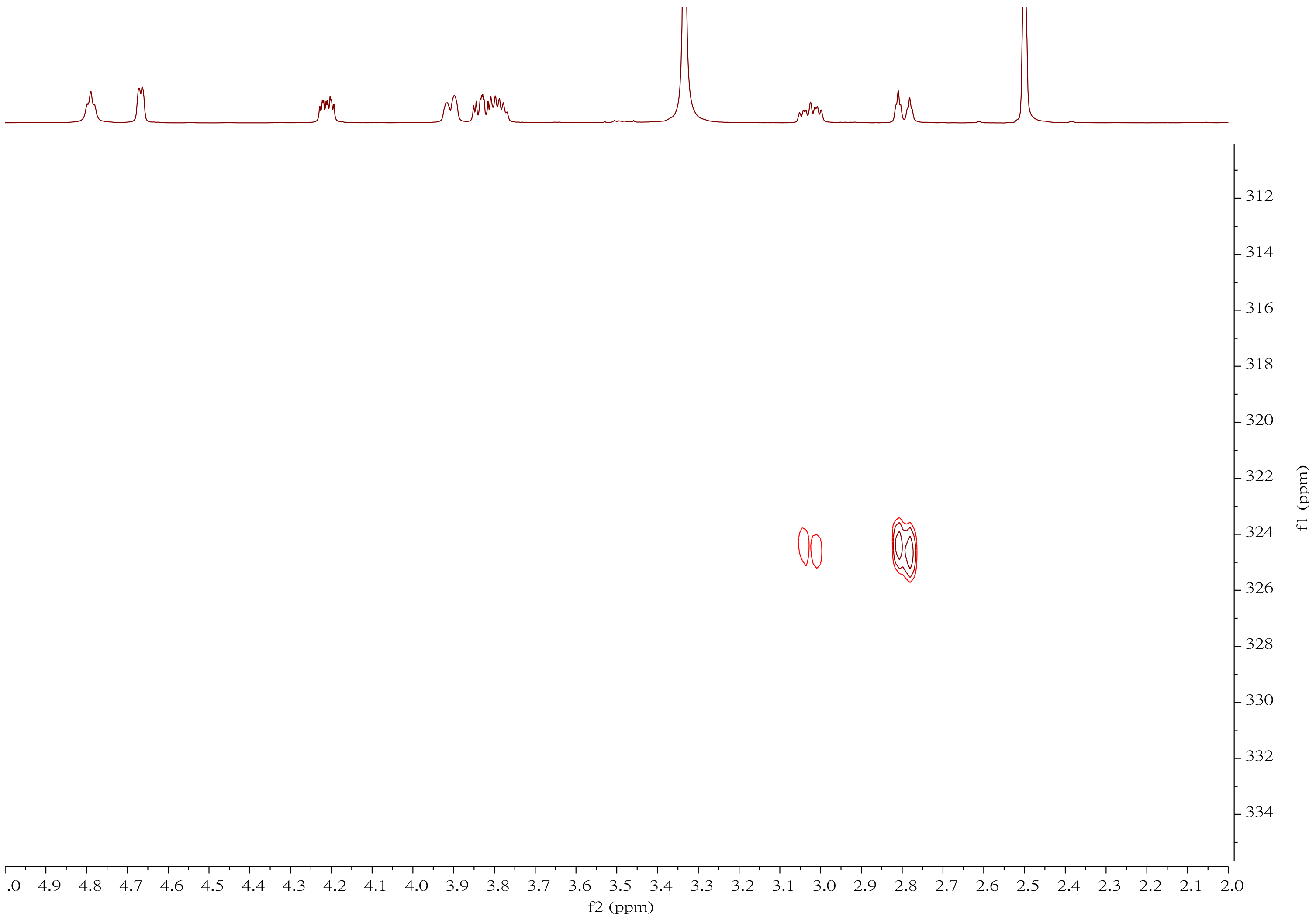

Figure S7. ^{1}H NMR spectrum of tuberazine B (**2**) (CD_3OD, 700 MHz)

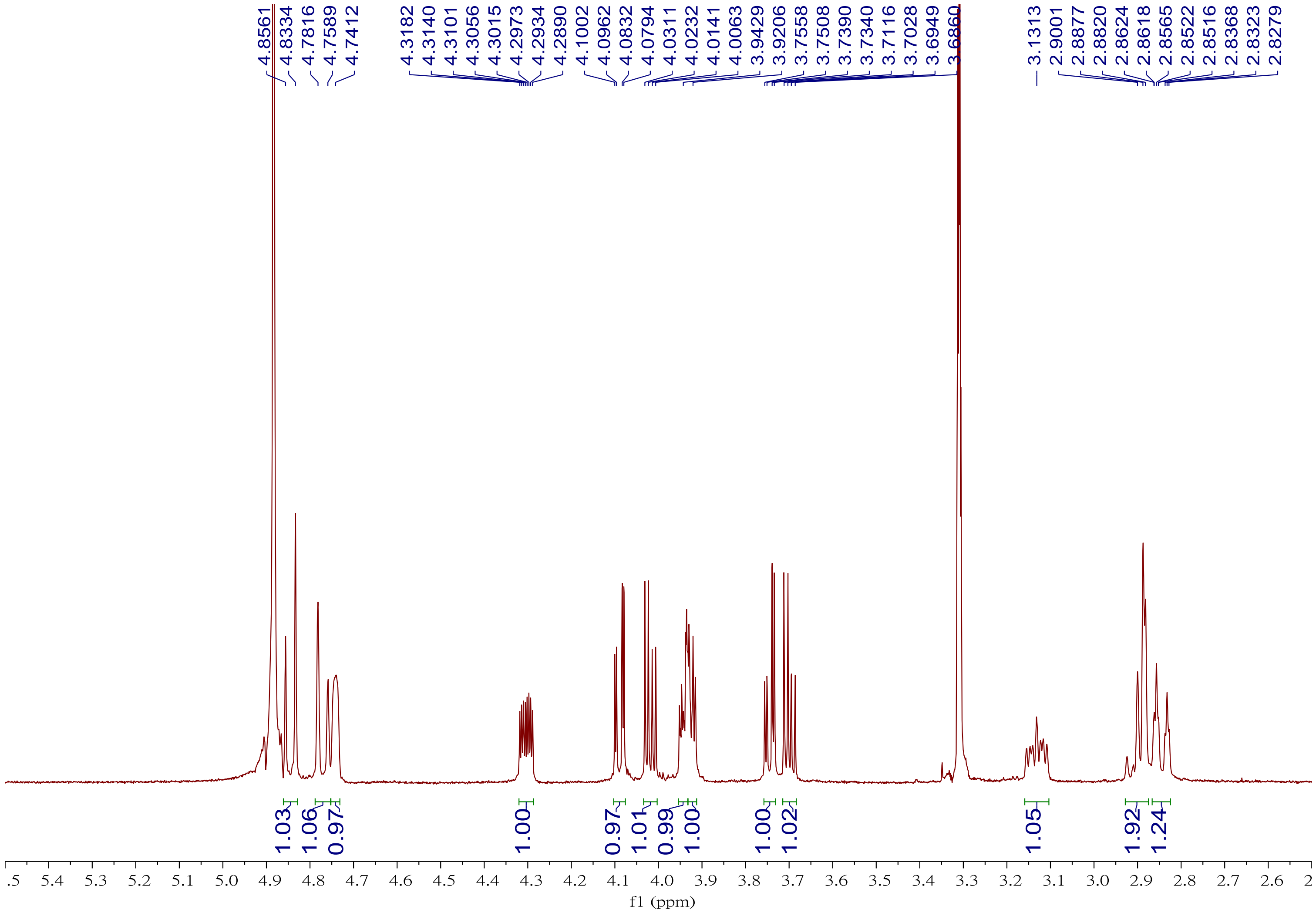

Figure S8. ^{13}C NMR spectrum of tuberazine B (**2**) (CD_3OD, 175 MHz)

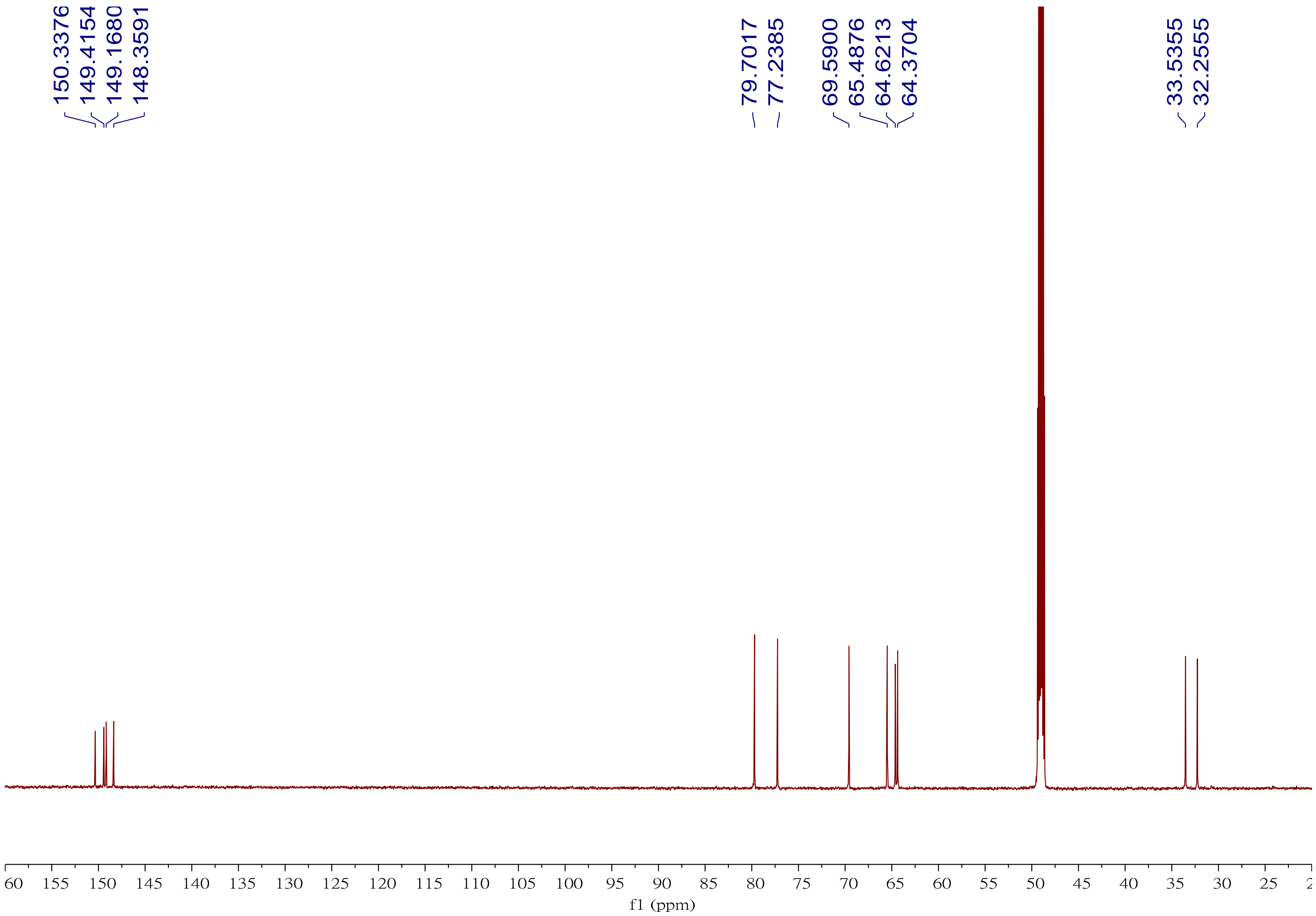

Figure S9. COSY spectrum of tuberazine B (**2**)

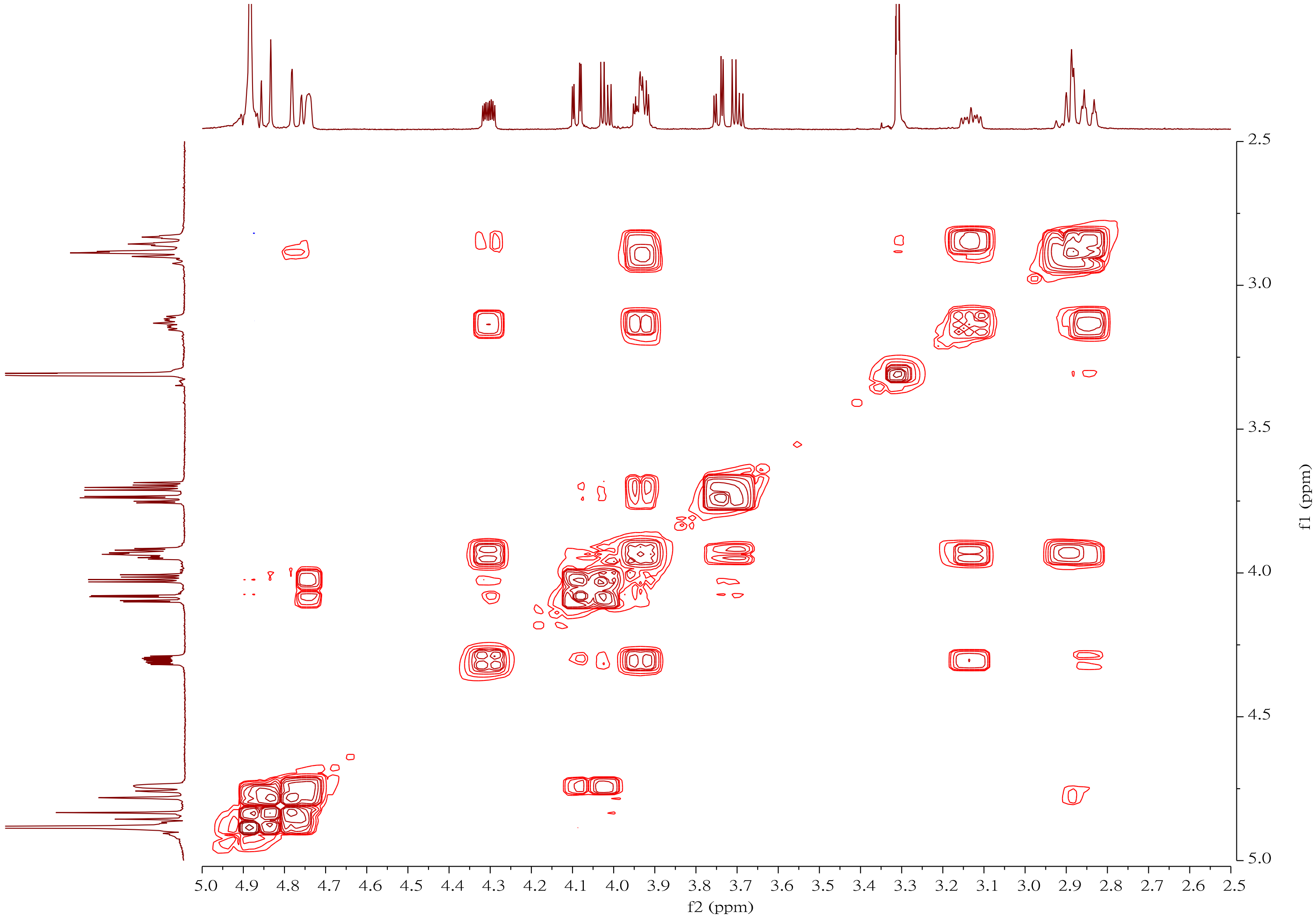

Figure S10. HSQC spectrum of tuberazine B (**2**)

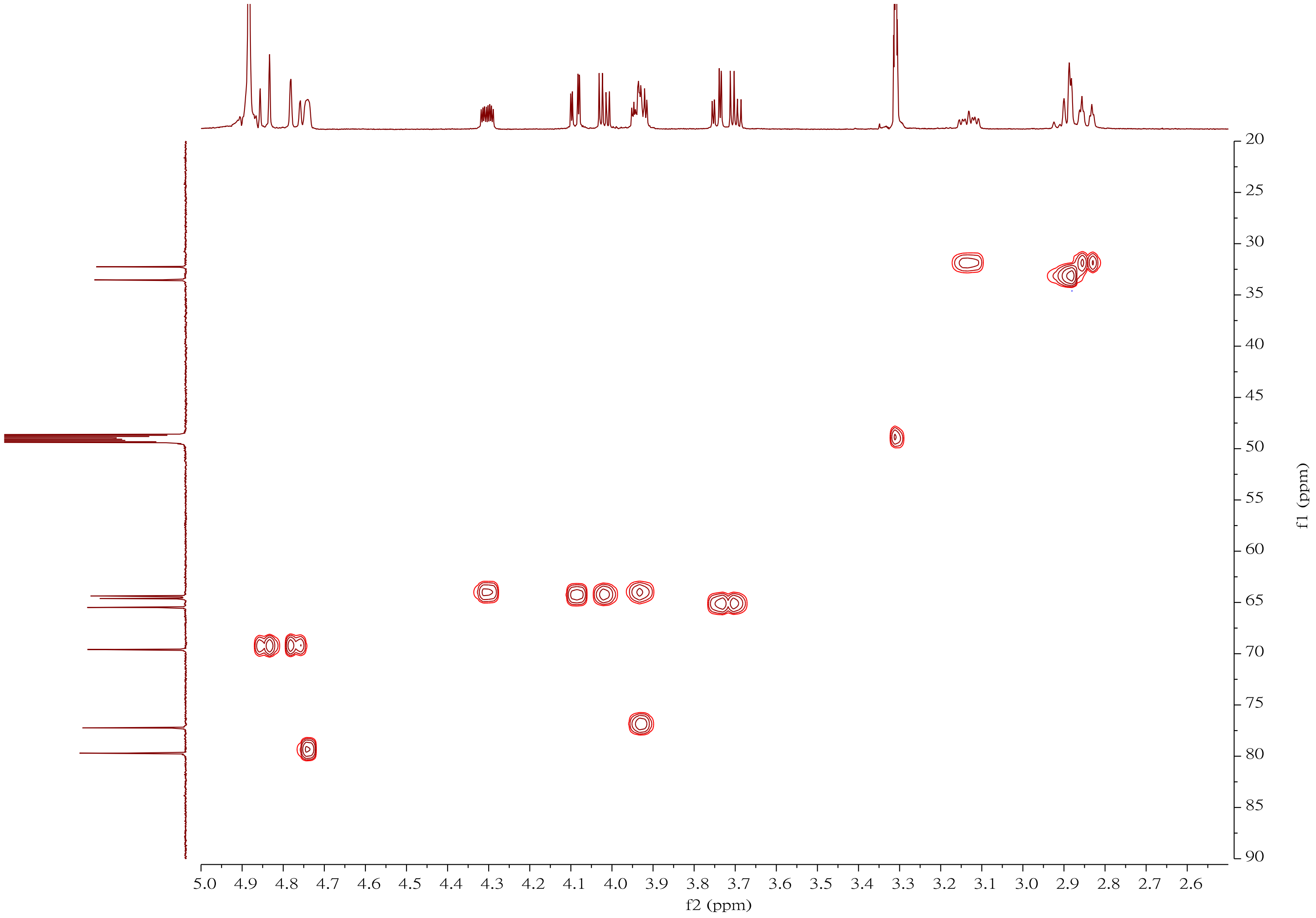

Figure S11. HMBC spectrum of tuberazine B (**2**)

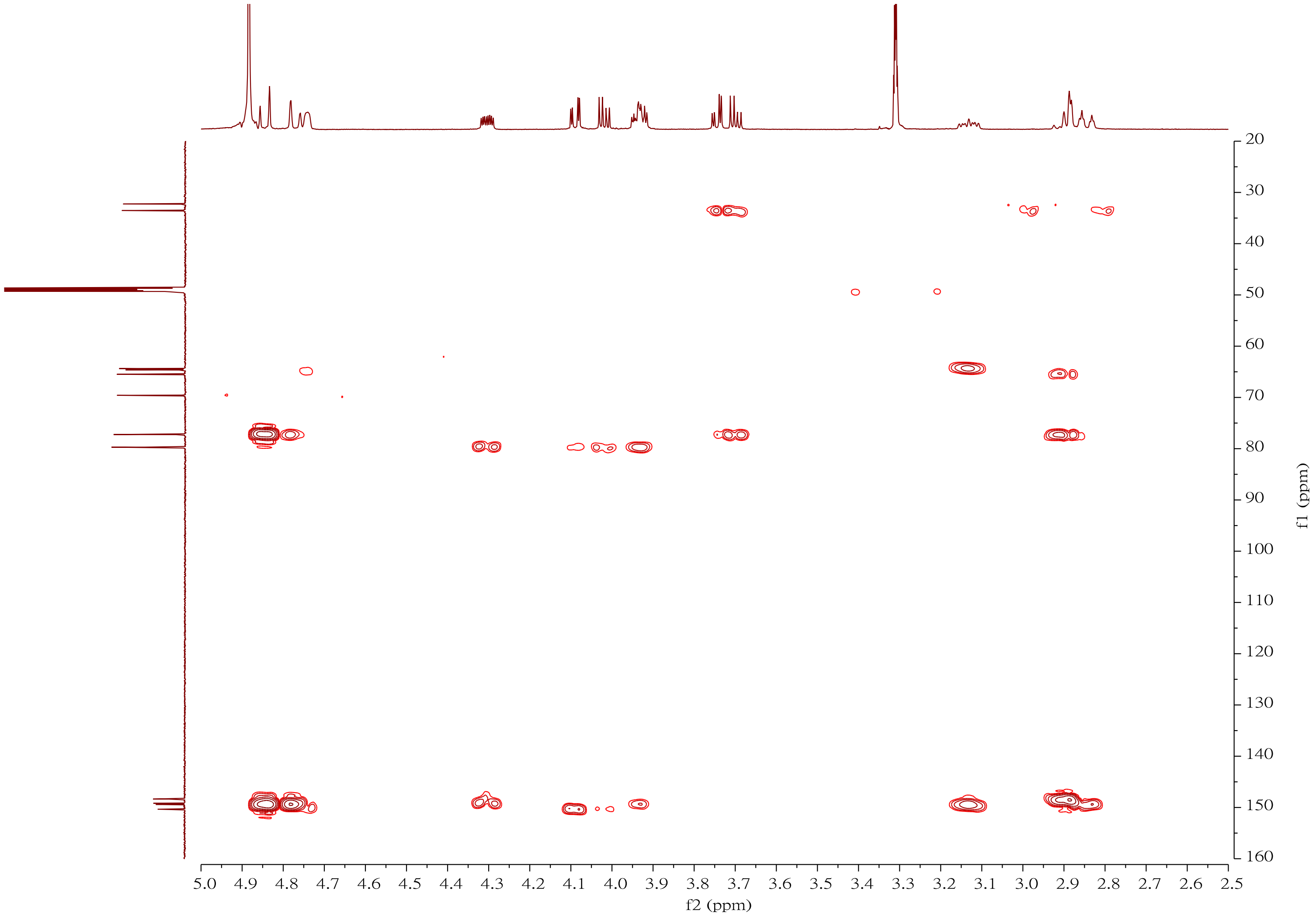

Figure S12. ^{1}H-^{15}N HMBC spectrum of tuberazine B (**2**)

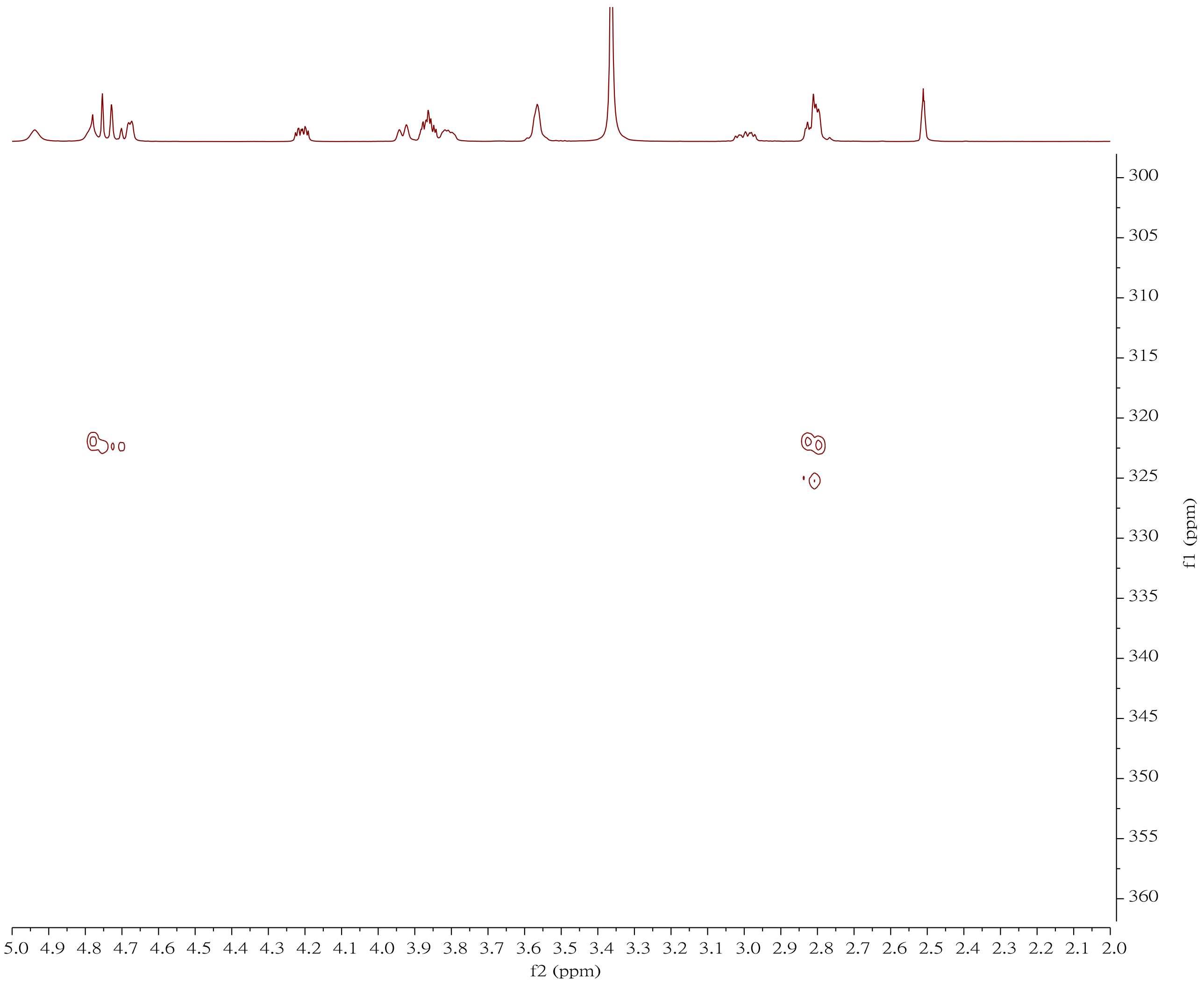

Figure S13. ^{1}H NMR spectrum of tuberazine C (**3**) (CD_3OD, 700 MHz)

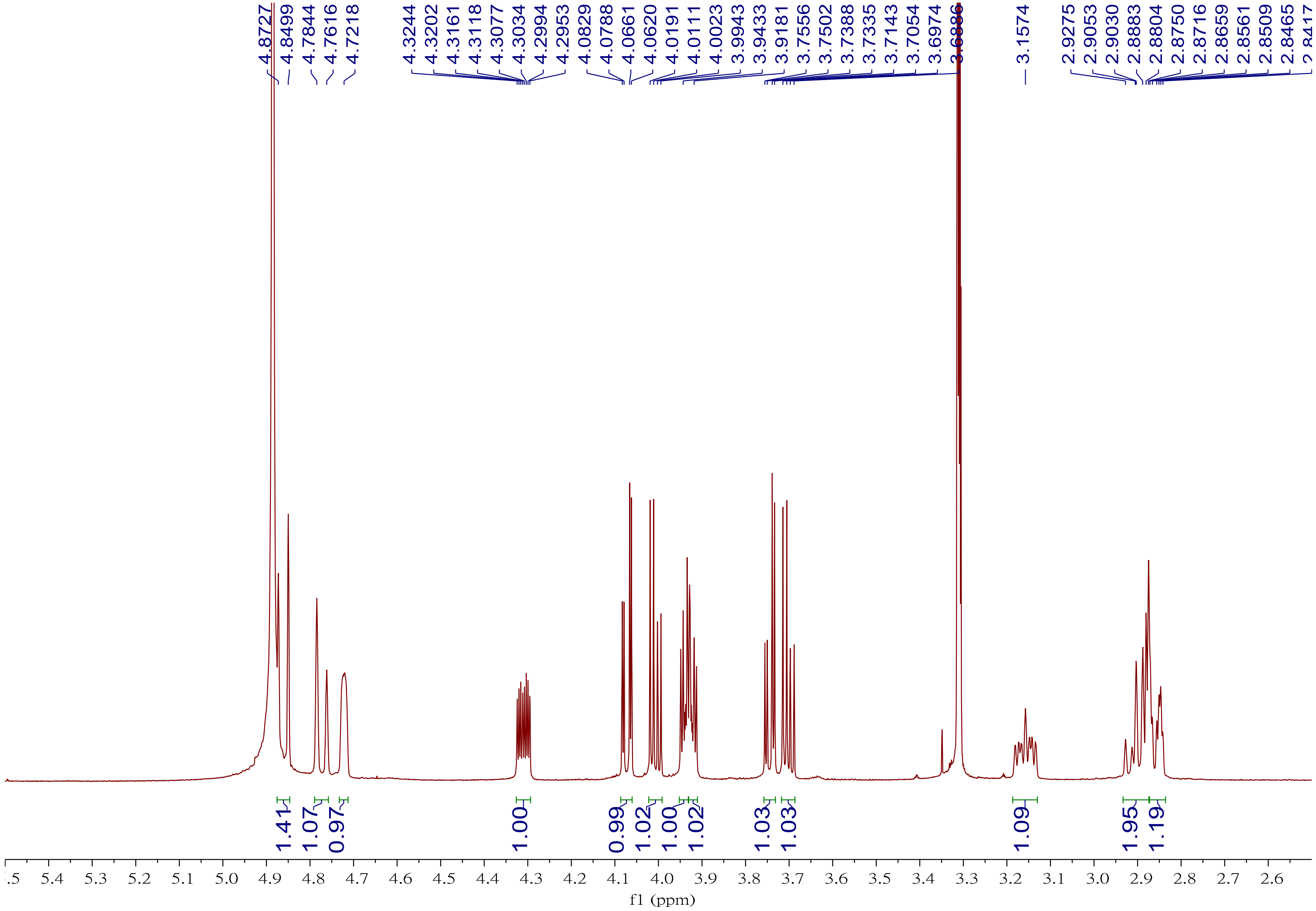

Figure S14. ^{13}C NMR spectrum of tuberazine C (**3**) (CD_3OD, 175 MHz)

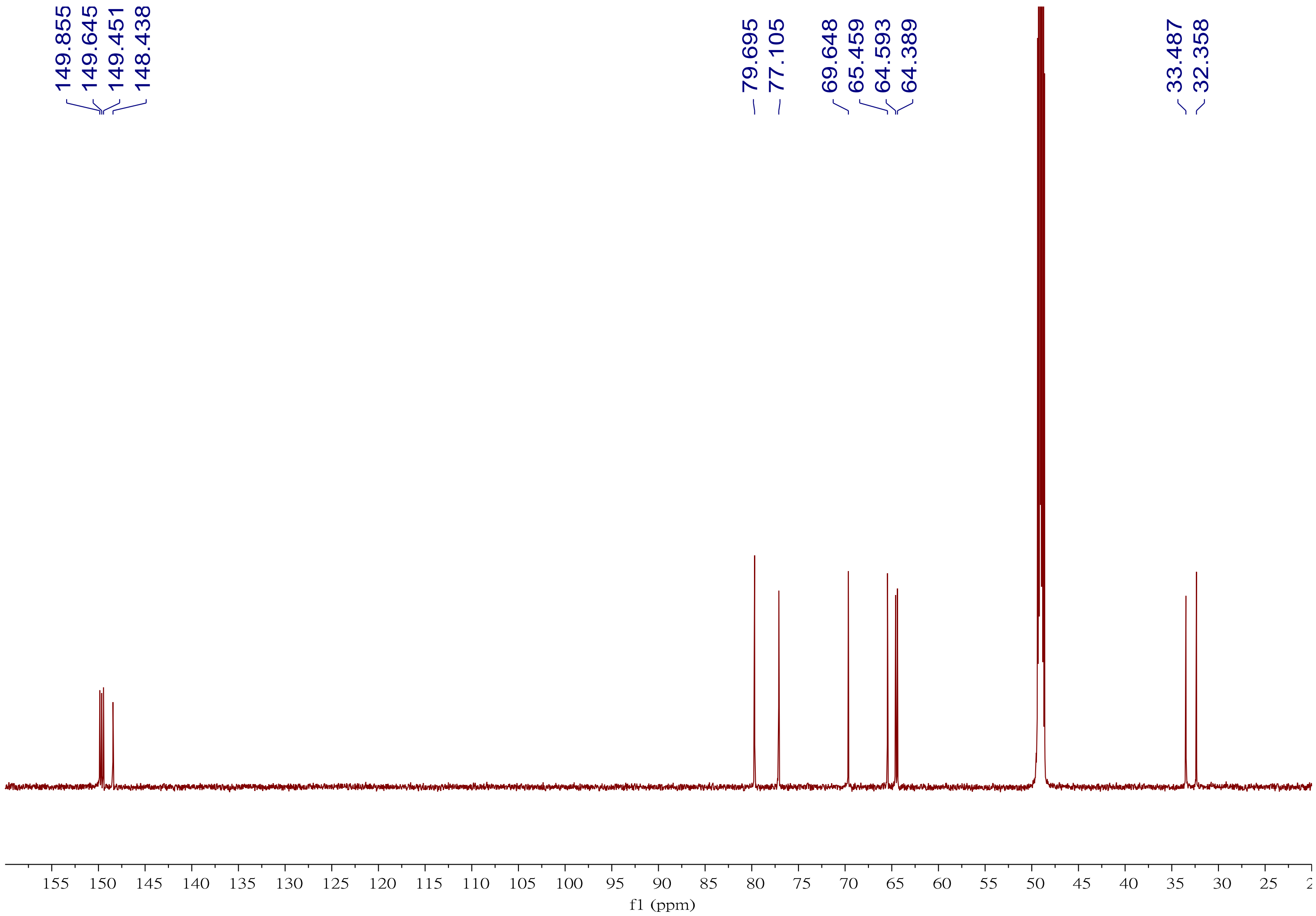

Figure S15. COSY spectrum of tuberazine C (**3**)

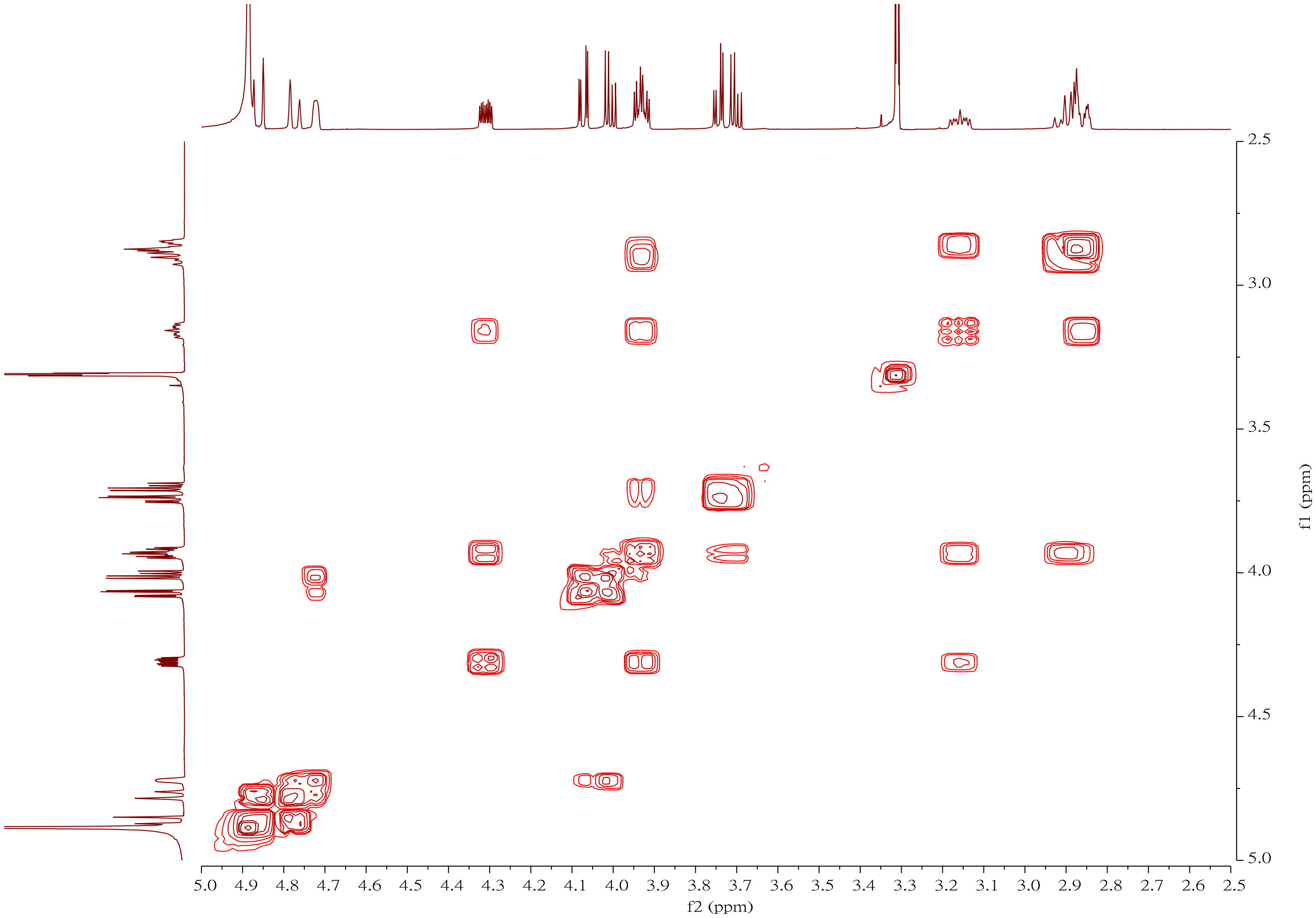

Figure S16. HSQC spectrum of tuberazine C (**3**)

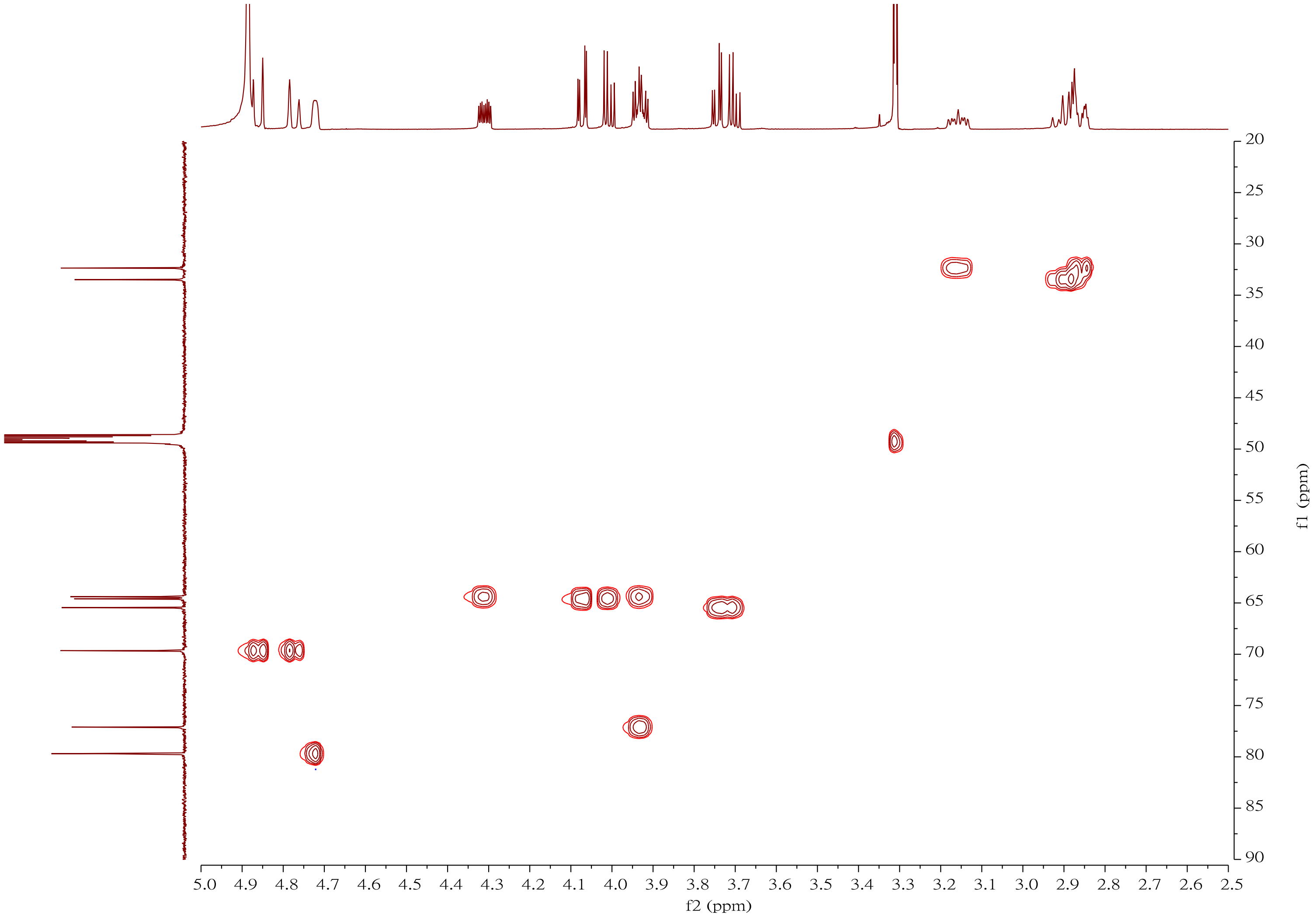

Figure S17. HMBC spectrum of tuberazine C (**3**)

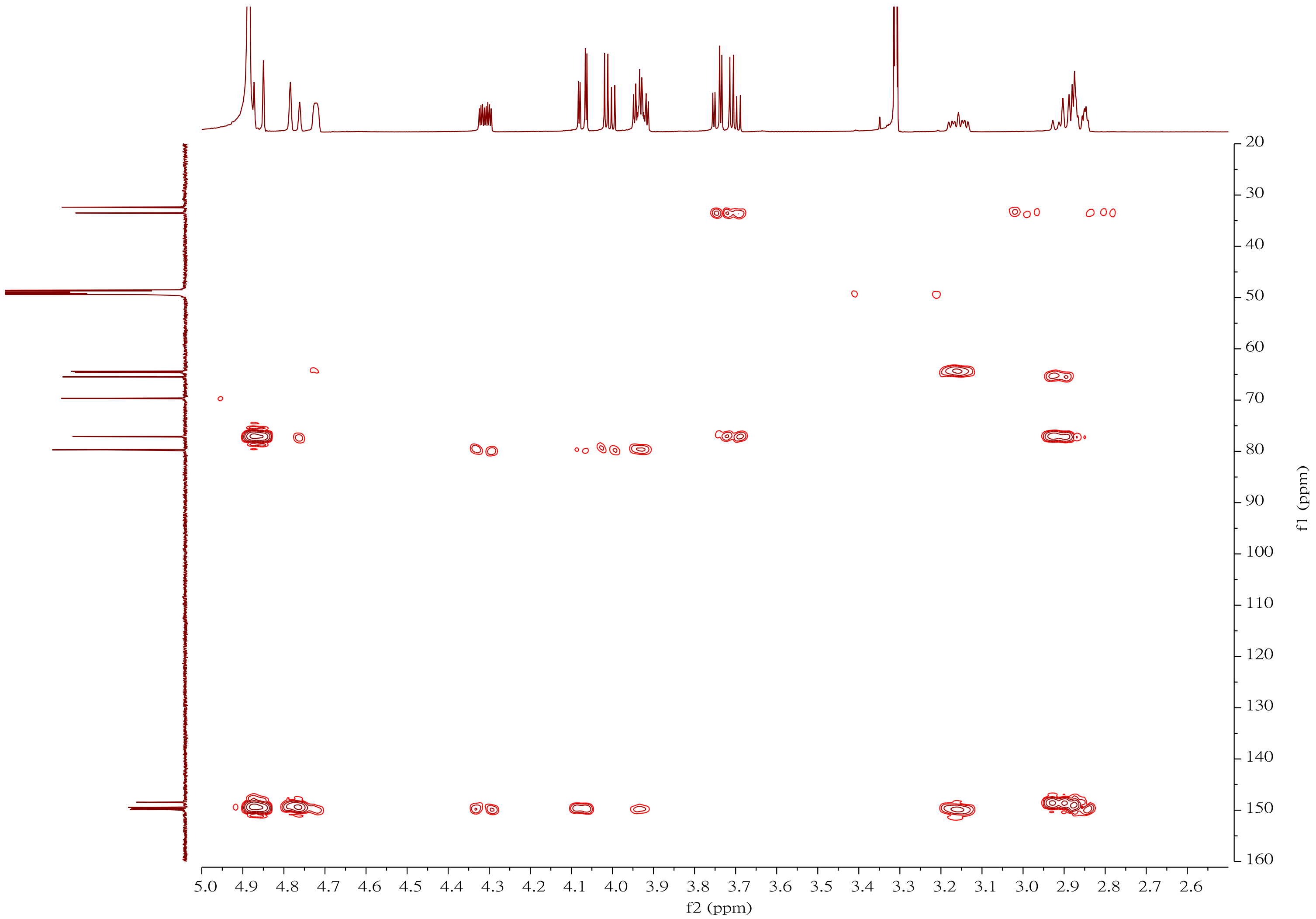

Figure S18. ^{1}H-^{15}N HMBC spectrum of tuberazine C (**3**)

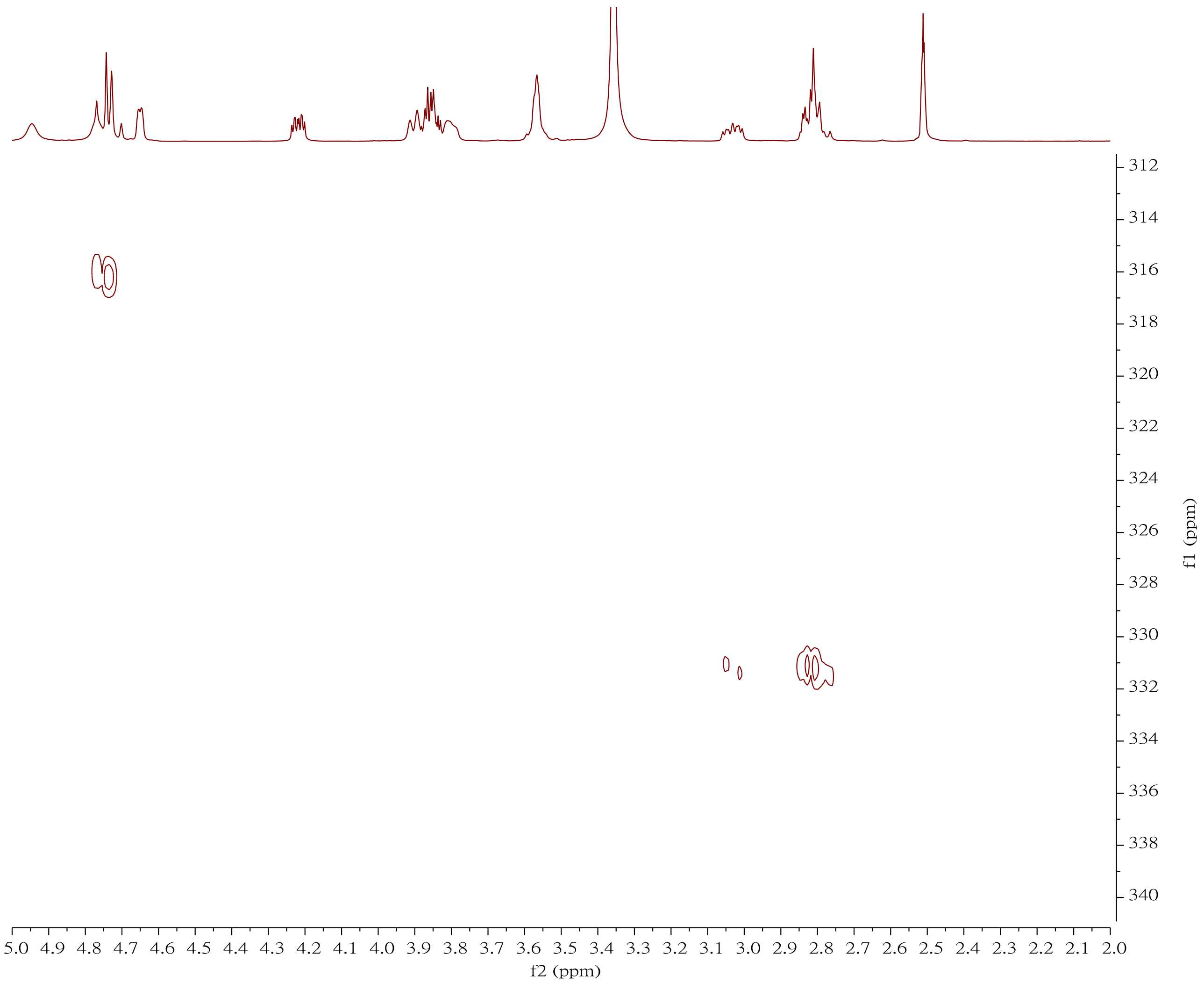

Figure S19. HRESIMS spectrum of tuberazine A (**1**)

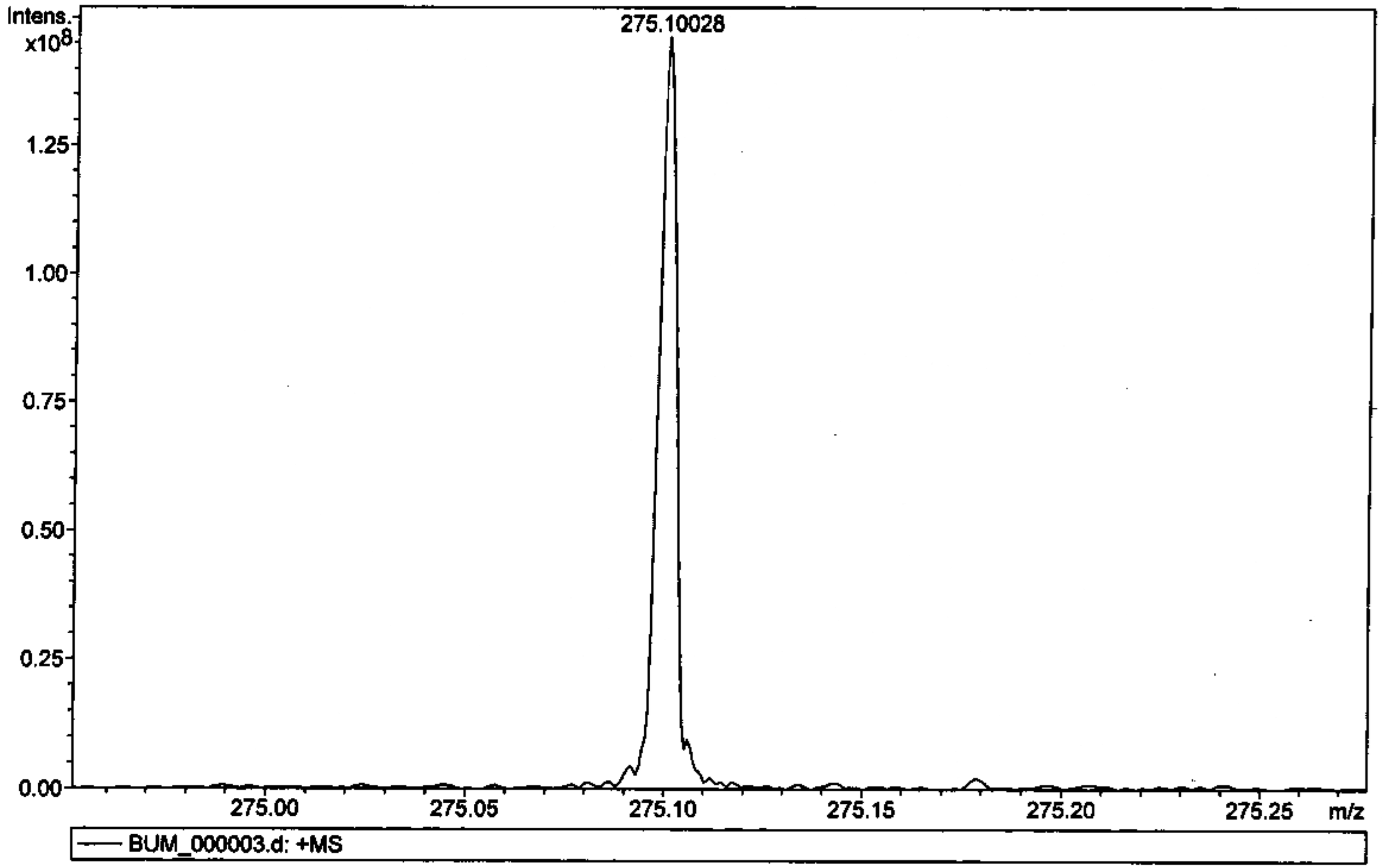

Meas. m/z	#	Formula	Score	m/z	err [mDa]	err [ppm]	mSigma	rdb	e⁻ Conf	N-Rule
275.10028	1	C 12 H 16 N 2 Na O 4	100.00	275.10023	-0.05	-0.19	7.3	5.5	even	ok

Figure S20. HRESIMS spectrum of tuberazine B (**2**)

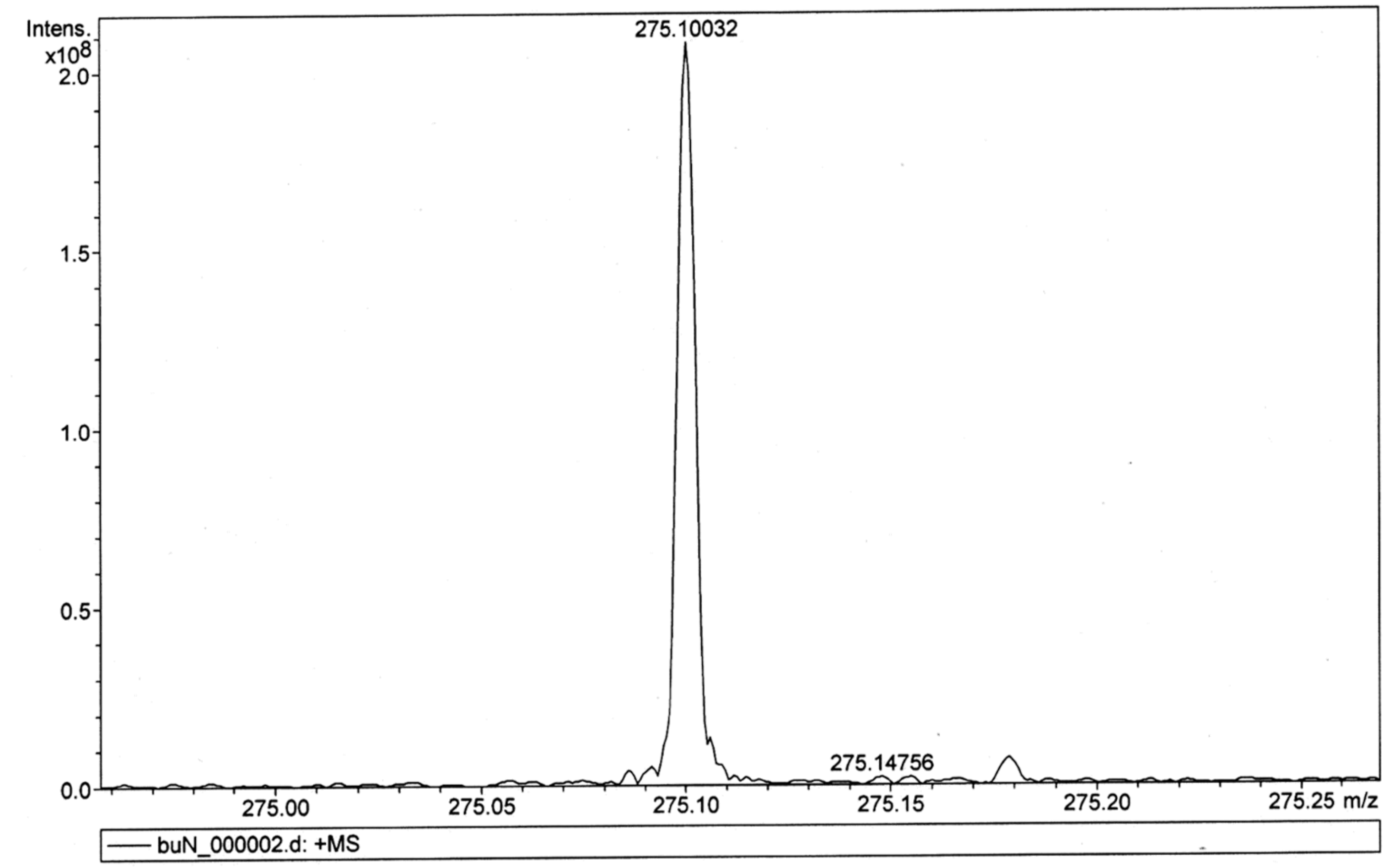

Meas. m/z	#	Formula	Score	m/z	err [mDa]	err [ppm]	mSigma	rdb	e⁻ Conf	N-Rule
275.10032	1	C 12 H 16 N 2 Na O 4	100.00	275.10023	-0.09	-0.34	2.6	5.5	even	ok

Figure S21. HRESIMS spectrum of tuberazine C (**3**)

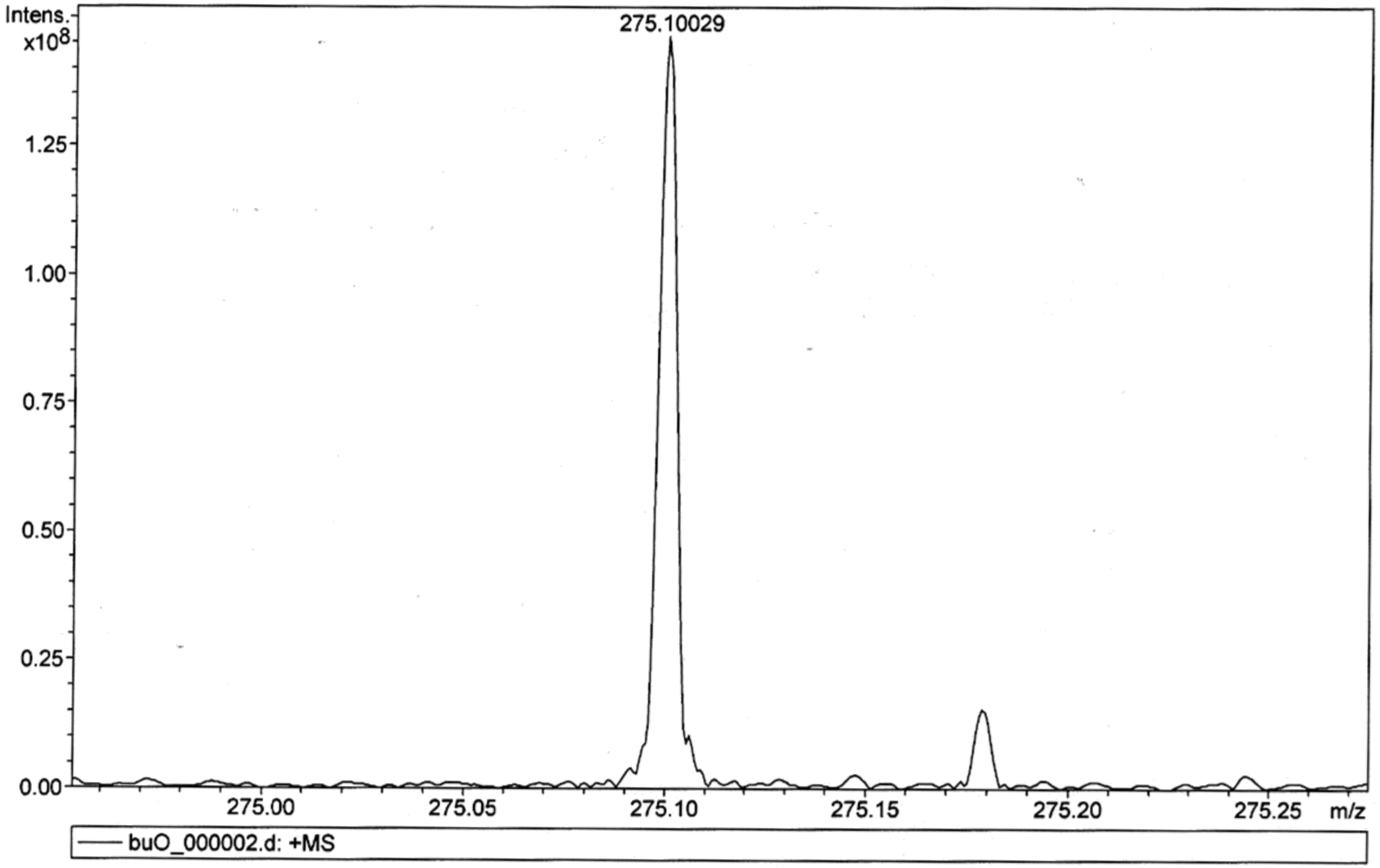

Meas. m/z	#	Formula	Score	m/z	err [mDa]	err [ppm]	mSigma	rdb	e⁻ Conf	N-Rule
275.10029	1	C 12 H 16 N 2 Na O 4	100.00	275.10023	-0.06	-0.22	3.7	5.5	even	ok

Figure S22. Possible structures of **1**

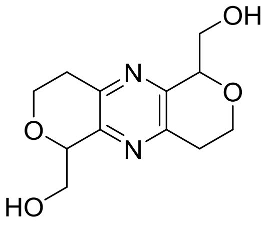

1

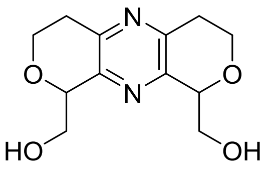

1a

Figure S23. Possible structures of **2**

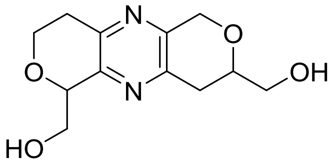

2

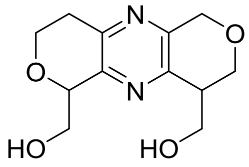

2a

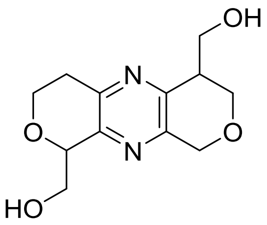

2b

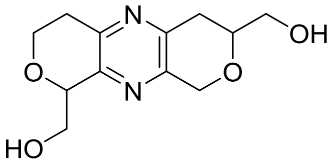

3

Table S1. Anti-lymphangiogenic activity of selected compounds

Compound	IC_{50} (μg/ml)[a]
Tuberazines A (**1**)	40 ± 2
Tuberazines B (**2**)	39 ± 2
Tuberazines C (**3**)	33 ± 1
Rapamycin[b]	< 5

[a]Half maximal cytotoxicity concentration. [b]Positive control.
